# Light Microscopy of Medium-Density Rigid Polyurethane Foams Filled with Nanoclay

**DOI:** 10.3390/polym14061154

**Published:** 2022-03-14

**Authors:** Ilze Beverte, Ugis Cabulis, Janis Andersons, Mikelis Kirpluks, Vilis Skruls, Peteris Cabulis

**Affiliations:** 1 Institute for Mechanics of Materials, University of Latvia, 3 Jelgavas St., LV-1004 Riga, Latvia; janis.andersons@pmi.lv (J.A.); skruls@pmi.lv (V.S.); 2 Latvian State Institute of Wood Chemistry, 27 Dzerbenes St., LV-1006 Riga, Latvia; ugis.cabulis@kki.lv (U.C.); mikelis.kirpluks@kki.lv (M.K.); peteris@ritols.lv (P.C.)

**Keywords:** polyurethane foams, medium-density, nanoclay, light microscopy, structure, dimensions, probability density function

## Abstract

Practical applications and mathematical modelling of the physical and mechanical properties of medium-density rigid polyurethane foams require knowledge of their structure. It is necessary to determine structural characteristics without destroying the foams and measuring each element. A methodology is described for the use of light microscopy on environmentally sustainable, medium-density rigid polyurethane foams (in the density region of ≈210–230 kg/m^3^), by the analysis of two types of light microscopy images: (1) Cutting surface images; and (2) Through-cutting surface images. The dimensions of structural elements of polyurethane foams, filled with the nanoclay Cloisite-30B at concentrations of 0.0%, 0.25%, 0.50%, 1.0%, 2.0%, 3.0%, and 5.0% from the mass of the filled reacting mixture, are estimated. Probability density functions of projections of bubbles’ diameters and struts’ length are determined using images in three mutually perpendicular planes. A mathematical model is developed for the restoration of the actual dimensions of bubbles’ diameters using data of cutting circles’ diameters. Intercalation and exfoliation of the filler’s Cloisite-30B mono-layers is evaluated via the basal spacing by X-ray diffraction at a 5 wt.% concentration of nanoclay.

## 1. Introduction

Medium-density rigid polyurethane (PU) foams is a cellular polymer applied as a structural material in various engineering solutions, especially in the automotive industry for test milling, design studies, and modelling, and as substructures for model pastes when making simple negative moulds and laminating moulds, etc. [1,2,3,4]. The foams are also used as encapsulants for electronic components to mitigate harsh thermal and mechanical environments as well as to provide electrical isolation [5]. The practical applications of such PU foams materials, e.g., from the industrial scale producers Sika JSC (Baar, Switzerland), General Plastics Manufacturing Company (Tacoma, WA, USA), Utah Foam Products, Inc. (Salt Lake City, UT, USA) etc., as well as the mathematical modeling of their physical and mechanical properties, require knowledge about foams’ structure.

A number of “Structure-mechanical properties” theories, based on (a) model-cells of different geometry, space filling, or un-filling [1,3,5,6,7,8], (b) methods of orientational averaging, operating with a singular “Strut-nod” element [9,10], and the (c) Voronoi approach when a series of random structures is generated and the mechanical behaviour is analyzed with the support of finite element models [11], exist for PU foams with an expressed strut-like structure (Density ρ_f_ < 150 kg/m^3^), but a shortage of modeling approaches for the structure of medium-density foams is revealed. A methodology for the restoration of the spatial structure of the strut-like structure by means of light microscopy images, taken in three mutually perpendicular planes, was described previously [12]. The distribution of struts’ length projections and the length themselves fits to a power law with an exponential cut-off, where the distribution of the struts’ angle projections fits to an exponential law. In [12,13], the differences between the projections and elements themselves (length and angles) were considered for the range of the most widely occurring elongation degrees of the PU foams’ cells. In principle, the methodology described in [12,13] is also applicable to medium-density cellular plastic where the polymeric struts can still be distinguished.

Methods have also been developed for describing the structural characteristics of high-density foams with unconnected bubbles imbedded in a continuous polymeric matrix [1,4,14,15]. An SEM study of rigid PU foams showed that at densities ρ_f_ = 380–1000 kg/m^3^, even in free expansion, the cells essentially appear spherical and closed. Their real diameters are determined and proved to follow a Gaussian distribution. The main principles of statistical analysis of the macrostructure of cellular plastics and mathematical models for finding the probability density functions of bubbles’ diameters based on experimentally determined distributions of cutting circles’ diameters are analyzed in [4] for low-porosity (<30%) plastic, which is comprised of isolated spheres. A lack of mathematical models and experimental data on the structure for PU foams of medium density is reported [1,2,3,4].

The image-based approach to microstructure investigation, 3D numerical modeling, and the prediction of foams’ mechanical behavior applies the most recently available imaging techniques: X-ray computed tomography (CT) and solid state nuclear magnetic resonance. The acquired set of images serves as a basis to recreate the geometry within a finite element code. The image-based approach considers the actual structure of the foams under investigation; however, it is at the price of very high computational costs and expensive research appliances [11,16]. In [11], an image-based approach was presented to describe the internal microstructure of 80, 100, 130, and 320 kg/m^3^ PET polymeric foams inspected with high-resolution CT. The imaged structure was converted into a finite element mesh to perform the analyses. In [16], the fracture toughness of PU foams of the low densities of 100 kg/m^3^ and 145 kg/m^3^, and of a high-density (300 kg/m^3^) PU foams, was analyzed in connection to the microstructure. The medium-density (≈200–250 kg/m^3^) PU etc. foams’ materials, featuring a transition from a strut-like structure to that of isolates gaseous inclusions in a monolithic polymeric matrix, are insufficiently covered by investigations.

Elasticity moduli and strength in compression and tension of rigid, closed-cell PU foams of a medium density of 195 kg/m^3^, with a space filling coefficient P1 = 15%, were determined experimentally in [17]. Mathematical modeling and numerical FEM calculations of the structure were made for increasing volume of nodes, decreasing length of struts, and thickening walls, joined into a cuboctahedron model cell. Light microscopy images are presented together with SEM images of the cutting surfaces, but no experimental data on the dimensions of structural elements was given.

Distribution graphs of cells’ diameters and their average values were determined by SEM microscopy of nanoclay-filled PU foams of a medium density of 200 kg/m^3^ in [18,19]. It was shown that the addition of nanoclay into a rigid PU foams’ structure did not change the isotropic character of the cellular structure and provided an improvement of physical and mechanical properties. The addition of Cloisite-15A or Cloisite-30B into a rigid PU foams’ matrix decreased the average cell size from 188 to 113 µm for the neat rigid PU foams and a 2.68 wt.% nanoclay content, respectively. The cell size decreased and the cell number in a unit volume increased with an increase of the nanoclay’s concentration in a similar mode for Cloisite-15A and Cloisite-30B fillers. It is emphasized that for SEM microscopy, imaging mainly the gold-coated, cut bubbles and walls, gives no clear understanding of the shape and dimensions of the other structural elements, such as the struts, nodes, walls’ cross sections, etc., as well as provides insufficient information on the spatial interconnection of elements.

Exfoliating montmorillonite (Mt.) to nanolayers is a crucial step during producing clay/polymer nanocomposites [20,21]. Only well-exfoliated and well-dispersed Mt. nanolayers in the polymer matrix can significantly improve the properties of the nanocomposites. The direct exfoliation of Mt. dispersed in water or organic solvents is often intensified by ultrasonication. The grinding of Mt. in the form of a solid in a high-energy ball mill can directly exfoliate Mt. to some extent. Exfoliating Mt. for producing clay/polymer nanocomposites is mainly achieved through the so-called in situ exfoliation, solution exfoliation, and melt exfoliation. The Mt./polymer nanocomposites exhibit improved barrier properties, mechanical strength, thermal stability, and fire retardancy [20,21].

Compared to limitations of other methods, light microscopy has several advantages: no expensive equipment is required and the size of selections of elements can be increased practically without limitations in order to increase the quality of estimations. The selections from several locations of a sample can be merged to characterize the cellular material more generally; in addition, the computerized processing time of the selections is reasonable. Therefore, adequate methods that take into account the peculiarities of medium-density rigid PU foams’ structure have to be developed for light microscopy.

Taking into account the wide industrial applications of medium-density PU foams and the identified shortage of reliable experimental data on their structure, as well as the limited information regarding the foams’ structure given of the available SEM data, the aim of this research is a light microscopy investigation with the aim of outlining of the main structural characteristics of medium-density rigid PU foams in a density range of ~210–230 kg/m^3^, both neat and filled with nanoclay. An experimental method is developed for identifying and characterizing PU foams’ structural elements in conditions when a large part of the images is covered by the cut bubbles. A mathematical model is described for determining the actual dimensions and probability density functions of the bubbles’ diameters. Other structural elements are outlined and their dimensions are estimated. The influence of a nanoclay filler on the structure of PU foams is estimated and conclusions are made.

## 2. Materials and Methods

### 2.1. Raw Materials for the Production of PU Foams 

The aromatic polyester polyol (APP) NEOpolyol-380 used in this study was produced by Neo Group, Klaipėda, Lithuania. The main business of the company is the production of PET granules and PET bottles. The side stream of those commodities comprises PET dust and other industrial waste, which is directly transferred into a glycolysis reactor where it is converted into APP. Higher functional polyether polyol based on sorbitol Lupranol 3422 (contains only secondary hydroxyl groups, OH value 490 mg KOH/g) from *BASF* was added to increase the cross-linkage density of the polymer matrix. An additive surfactant, NIAX Silicone L6915, was used to obtain closed-cell PU foams. The reactive delayed action time amine-based catalyst NP-10, available from Momentive Performance Materials Inc., was used. Tris (chloropropyl)phosphate (TCPP) from LANXESS Deutschland GmbH was used as a flame retardant. Distilled water was used as a chemical blowing agent, thus ensuring foaming without the emission of harmful, ozone-depleting substances that might contribute to climate change. Polymeric diphenylmethane diisocyanate—IsoPMDI 92140 (pMDI) from BASF, Ludwigshafen, Germany—was used as an isocyanate component (NCO = 31.5 wt.%).

Commercially available montmorillonite nanoparticles, Cloisite-30B (manufacturer BYK Additives), were used. Cloisite-30B is organically modified with the organic modifier methyl, tallow, bis-2-hydroxyethyl, quaternary ammonium, which has a specific gravity of 1980 kg/m^3^. The size distribution of clay agglomerates prior to dispersion were ~50% ≤ 6 μm (6000 nm) and ~90% ≤ 13 μm (13,000 nm), with X-ray diffraction d-spacing (001) 18.5 Å.

### 2.2. Production of PU Foams in a Sealed Mould

Since medium-density rigid PU foams’ industrial items are often produced in sealed moulds, rigid polyurethane (PU) foams’ blocks were made in a sealed steel mould that was shaped as a truncated pyramid, with the dimensions top 15 × 15 cm, bottom 14 × 14 cm, height 5 cm and inner volume V_0_ = 0.00105 m^3^. 

The formulation of the PU foams is given in Table 1.

The formulation is comprised of ~15% of recycled materials, being pure industrial PET waste, facilitating the production of environmentally sustainable materials. The nanoclay Cloisite-30B was added as a filler in concentrations of η = (1) 0.0, (2) 0.25, (3) 0.50, (4) 1.0, (5) 2.0, (6) 3.0, and (7) 5.0% from the mass of the filled reacting mixture by mixing into the NEOpolyol-380 with a high shear mixer from Silverson (East Longmeadow, MA, USA), for 20 min at 8000 rpm. For a comparative XRD analysis, a 5 wt.% Cloisite-30B dispersion was also made, applying the Ultrasonic Cell Crusher from MRC (Harlow, Essex, UK) over short periods, with a frequency of 20–25 Hz, for 5 s of an active period and 5 s of a passive period (to reduce any heating of the mixture), resulting in a 20 min effective time. The temperature limit 80 was set to 40 °C and was controlled by a water bath. At both dispersion modes, the result was a milky, homogenous, stable dispersion.

In the given research, PU foams are considered as medium density up to ~250 kg/m^3^. Light microscopy showed that at a density of ~300 kg/m^3^, the structural elements-polymeric struts already can’t be outlined and the structure is formed by isolated gaseous bubbles in a polymeric matrix. The lower density boundary of medium-density foams can be defined as that at which the struts have average longitudinal dimensions exceeding the average transversal dimensions no more than 1.3–1.5 times, which corresponds to densities of ~150–170 kg/m^3^.

The mass of the liquid reacting mixture, poured into the mould, was calculated so as to ensure that the PU foams’ blocks had an apparent overall density of ~250 kg/m^3^ (ISO 845:2006), Figure 1. The technological target was to keep the mass of the filled reacting mixture for all seven concentrations constant: m_0_ = 250 g = const. For the actual blocks made, deviations in their mass may appear during the technological procedures due to: (a) the filled reacting mixture remaining on the inner walls of the mixing vessel, (b) uncertainty of the scales, (c) a different mass of reacting mixture escaping through gas-release holes due to the influence of the nanoclay’s concentration on the mixture’s viscosity, and (d) a subjective mistake of technologist, etc. Detailed information on the production of the PU foams is given in the Section S1 of the Supplementary Materials. Density in free rise of neat PU foams of the given formulation was determined as well, producing the foams in an open mould of the same lateral dimensions as those of the sealed mould. The content of closed cells in neat PU foams was determined according to ISO 4590:2016. The PU foams of the given formulation at an apparent overall density of ~200 kg/m^3^ exhibited competitive physical and mechanical properties [18,19].

### 2.3. The Microscopy Samples

**Figure 2:**
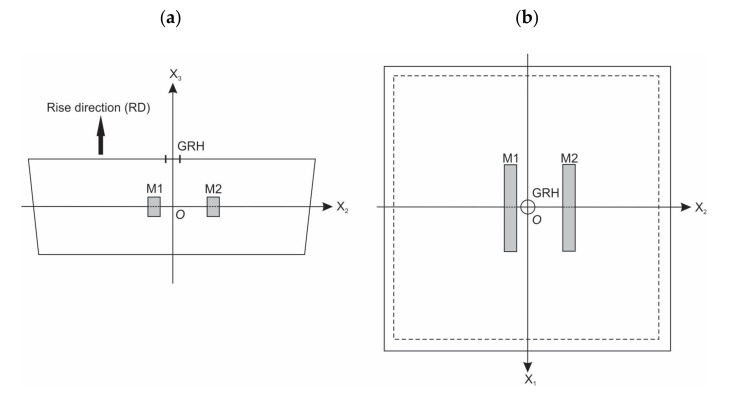
Microscopy samples, M1 and M2, from a PU foams’ block: (**a**) cross-sectional view (Plane X_2_OX_3_) and (**b**) top view (Plane X_1_OX_2_); GRH, the gas-release hole.

The placement of microscopy samples in a molded PU foams’ block is depicted in Figure 2. Two samples, M1 and M2, were cut from the central part of each of the seven molded PU foams’ blocks to avoid the foams’ higher density and modified structure due to adiabatic processes at the mould’s walls. The sample M1 was located at a distance of ~5 mm (one thickness) from the axis OX_3_ of the block and the sample M2 was at a distance of ~15 mm (three thicknesses) from OX_3_. The parallelepiped-shaped microscopy samples of dimensions 5 × 10 × 40 mm were oriented with surfaces parallel to the planes X_1_ = const., X_2_ = const., and X_3_ = const. of the Cartesian coordinate system, where axis OX_3_ is parallel to the rise direction and the plane X_3_ = const. is the plane of the foams’ isotropy (i.e., the plane X_1_OX_2_). The apparent core density ISO 845:2006 (further-density) was determined for each sample.

### 2.4. Methodology of Light Microscopy

Thin slices of foams were cut with a razor blade, with a thickness 0.10 mm, from three mutually perpendicular sides of each microscopy sample and fixed on a glass slide, marking the rise direction. The slices were cut as (a) parallelepipeds and (b) narrow-angled wedges. A light microscope Diamond MCXMP500, MICROS Produktions-&Handels GmbH, Austria, with processing software and calibration reticle was used for the investigation of the PU foams’ structure at magnification of 10×. Light microscopy of the seven samples of M1 showed an influence of the gas-release hole in the mode of (1) a skewed, untypical structural anisotropy due to a directed flow of escaping bubbles and (2) the presence of singular, untypically large bubbles. Therefore, the M1 samples were omitted and only the seven M2 samples, situated comparatively far from the gas-release hole, were further used for microscopy. More details of the methodology are given in the Section S2 of the Supplementary Materials.

**Figure 3:**
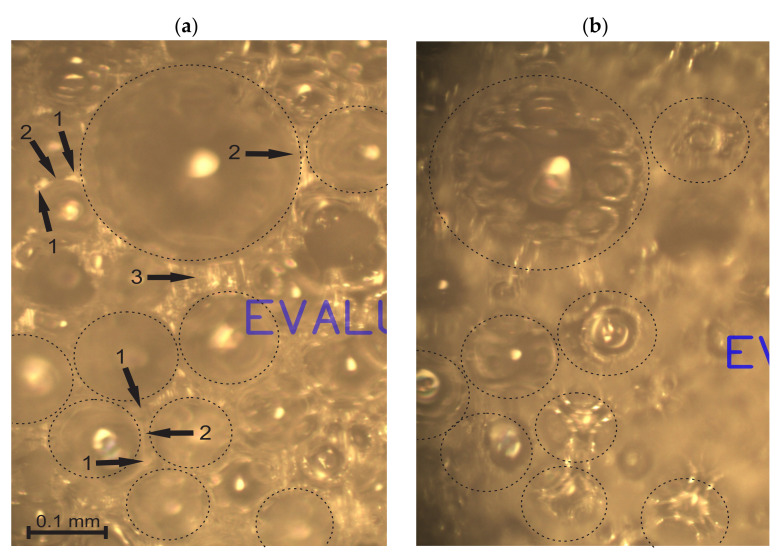
One and the same location of neat, medium-density PU foams, ρ = 211 kg/m^3^ (P1 = 16.5%): (**a**) cutting surface image with struts (1), walls (2), and un-foamed volumes (3); and (**b**) through-cutting surface image. Image plane X_1_ = const.

In the given samples of medium-density rigid PU foams with a density of 211 ≤ ρ_f_ ≤ 232 kg/m^3^ and ~99% of closed-cells, the dimensions and volumes of cells’ walls are comparable to those of struts and nodes. The methodology for a light microscopy investigation of such structures had to be described. At first, the focus was adjusted on *the cutting surface* of a slice and images were taken where the surface of the cut monolithic polymer was clearly visible, e.g., Figure 3a. The dimensions of gaseous inclusions (the bubbles), the thickness of walls, and the dimensions of un-foamed volumes were estimated from the cutting surface images.

**Figure 4:**
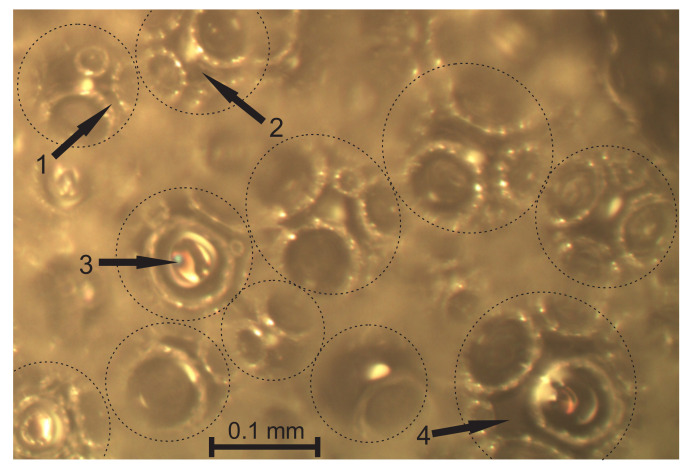
A through-cutting surface image of neat, medium-density PU foams, ρ_f_ = 211 kg/m^3^ (P1 = 16.5%), image plane X_1_ = const.: 1, a strut, 2, a node, 3, a front view of a wall, and 4, an un-foamed volume.

The struts, nodes, un-foamed volumes, and front views of the walls are seen dimly through the circular voids in the surface and can be neither identified nor measured precisely. Therefore, the focus was adjusted until the elements were visible sharply and *a through-cutting surface image* of the same location on the surface was taken, as shown in Figure 3b. The dimensions of the struts and nodes, as well as walls’ diameters, were measured from the through-cutting surface images. On the through-cutting surface images, the cut bubbles appear as dim contours with circular areas with sharp visibility of the underlying elements, as presented in Figure 4. That provides additional information, therefore allowing both kinds of images to be analysed in parallel. On wedge-shaped slices, the cutting surface and the through-cutting surfaces can be seen simultaneously.

Light microscopy of medium-density PU foams faces several challenges. The bubbles, walls, struts, nodes, and other structural elements have to be defined to choose them properly for statistical samples. For low-density PU foams, the criteria for inclusion into a statistical sample are clearer due to the pronounced shapes and dimensions (the strut-like structure where nearly the entire polymer is comprised in struts and nodes). The considered medium-density PU foams feature a mixed structure (un-slim struts, comparatively large nodes, thick, curved walls, and presence of un-foamed volumes) that marks a transition from a strut-like structure to that of isolated, spherical gaseous inclusions in a continuous polymeric matrix. Elements become complicated to define because: (1) the transversal dimensions of struts are comparable to the lateral, (2) on the cutting surface, images of the cross section of a wall between two bubbles often look like a strut, (3) the nodes are irregular and have dimensions comparable to those of the struts (Figure 4), (4) it is often hard to distinguish between a node, a wall, and an un-foamed volume, and (5) since projections of the struts and nodes are clearly visible only on the through-cutting surface images, where the dim, unfocused cutting surface covers a considerable part of the image, a number of images are needed to form a statistical sample of sufficient size. 

Elements on the images de facto are projections of the actual structural elements on the image plane. The projections were analyzed and dimensions measured from printed images, with the image files open on the PC display, taking into account the scale. A subjective tendency of the observer to (a) choose bigger elements and (b) to not identify or include smaller elements into the statistical sample was recognized and constantly corrected. The actual spatial dimensions were determined for the diameters of bubbles and the length of struts. For the other elements it was assumed that the dimensions of the projections differ little from the dimensions of the elements themselves.

#### 2.4.1. Bubbles

**Figure 5:**
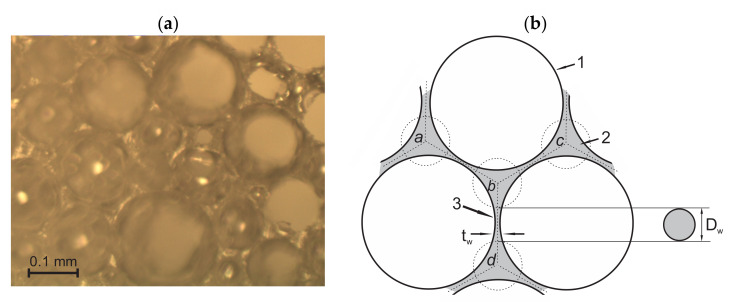
(**a**) Cutting surface of PU foams, ρ_f_ = 224 kg/m^3^, a wedge-shaped slice, with the inner side of the cut bubbles visible and (**b**) a scheme of cross-sections of 1—a bubble, 2—a strut (Transversal cross-section), and 3—a wall (t_w_—thickness and D_w_—diameter of the front view). Image plane X_1_ = const.

Elements of the foams’ structure are formed as the result of nucleation, expansion, merging, and stretching (in case of anisotropy) of gaseous inclusions—the bubbles, as presented in Figure 5. Diameters of the bubbles’ cross-sectional circles were measured from the cutting surface images. Images of slices cut from three mutually perpendicular sides, X_1_ = const., X_2_ = const, and X_3_ = const. (OX_3_ parallel to the rise direction), of each of the seven microscopy samples were taken and analysed. To gather statistical samples (selections) of sufficient size (200–250 typical elements), 3 to 5 images were necessary to be made for each cutting surface, at different locations.

A rectangular area (11.2 × 8.4 cm on an A4 format printed image; ~0.65 × 0.50 mm in nature) of a typical structure for the given concentration of Cloisite-30B was marked on each image, and the typical circles imaged on it were numbered and measured. If the diameter of a circle exceeded the average diameter of typical circles >2–3 times, the circle was considered as untypical and was disregarded. The diameters of 50–70 circles were measured from the printed images of each of the three cutting surfaces and grouped into three corresponding selections. The scale of the printed images (A4 format) was 1.0000:0.0057. For low-porosity (P2 < 30%) isotropic foams, the variation series of diameters, d, of un-interconnecting circles on the samples’ cutting surface are often approximated with probability density functions (PDF) in the mode of a power law with an exponential cut-off [4]:

f(d, q, b, α) = 1/A d^q^ exp(−αd^b^); (1)

d > 0; q, b and α > 0; f(d, q, b, α) ≥ 0.(2)

where q, b, and α are parameters determining the sharpness of maximum and asymmetry and A is a normalization factor. Due to the symmetric character of the experimental histograms, the normal PDF (the Gaussian distribution) was considered as well

(3) $$f\left(d,\mu ,\sigma ,\;\right)=\frac{1}{\sqrt{2\pi }\sigma }\;\exp\left[-\frac{{\left(d\;-\;\mu \right)}^{2}}{2\sigma^{2}}\right],\;$$

where μ is the mean and σ is the standard deviation. Both hypotheses of fitting between the probability determined from experimental data histograms f^E^(x) and theoretical approximating functions f^T^(x) were evaluated according to Kolmogorov’s criterion [13,22]:

(4) $$D_{n}=\max|f^{E}(x)-f^{T}(x)|\;\mathrm{and}\;D_{n}\sqrt{N}\;\geq \;\;\lambda ,$$

where N is the number of elements in the statistical sample and f(λ) is a tabulated probability for the quantity 0.0 ≤ λ ≤ 2.0 Although the histograms are slightly skewed to the bigger vales of diameters, the normal PDF showed a considerably better fitting than the asymmetric one and was further used for the approximation of experimental data histograms. To determine PDFs of the bubbles’ diameters f(D) based on PDFs of the diameters, d, of bubbles’ cross sectional circles, a mathematical model was described (Section 3.2). 

The number of bubbles per unit of volume of the foams was calculated from the following Equation [19]:

N_b_ = (nM^2^/A)^3/2^, (5)

where A is a rectangular area (in cm^2^) on the printed A4 format micrograph (taken at magnification M of microscope), where circles of cut bubbles were counted, n is the number of circles in the area A, and M is the linear magnification, as determined from a printed A4 format micrograph of the scaling reticle, taken at the same magnification M of the microscope as the micrograph of foams. Circles were counted on the same micrographs where their diameters were measured. The anisotropy coefficient was calculated as:

(6) Kaniz=1/n ∑i=1nd1id2i, 

where d_1_ and d_2_ are the dimensions of the bubbles’ cutting shapes in two mutually perpendicular directions. 

#### 2.4.2. Walls

A wall is a polymeric interface between two bubbles; each wall belongs to two bubbles [1,3,4,17]. The shape of the wall’s front view and the wall’s diameter, shown in Figure 5, were estimated from the through-cutting surface images. The wall’s thickness in the centre and along the perimeter was determined from printed images of three cutting surfaces. The scale of the printed images was taken into account.

#### 2.4.3. Nodes

A node is an un-foamed polymeric volume entered by ~4–6 struts [1,3,6,7,8]. The dimensions of a typical node should not exceed the transversal dimensions of a typical strut more than ~1.5–2.0 times. Un-foamed polymeric elements of larger dimensions are considered as un-foamed volumes. The nodes are modelled with spheres of diameter d_n_. The projections of the nodes were determined from the same through-cutting surface images as the projections of struts. A circular contour was drawn around each node on the printed image and its diameter was measured, taking into account the scale. United selections of 100–150 projections of diameters, d_np_, on the three image planes X_1_ = const., X_2_ = const., and X_3_ = const. were formed for each microscopy sample. It was assumed that the projections of the nodes’ diameters differ little from the diameters themselves: d_np_ ≈ d_n_. 

#### 2.4.4. Length of Struts

**Figure 6:**
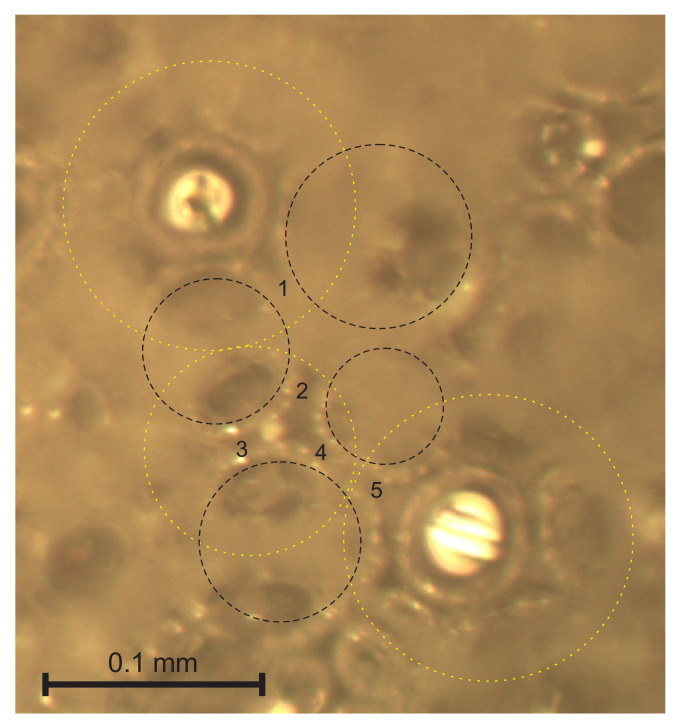
Struts 1–5, each formed between three bubbles, being an upper bubble (yellow) and two lower ones (black).

A strut is a polymeric element, formed by the flow of the liquid reacting mixture through Gibbs channels formed by the contact of three bubbles [2,4]. On images of cutting surfaces, the cross-section of a wall can’t be distinguished reliably from a longitudinal cross-section of a strut. Only struts from the through-cutting surface images were considered for inclusion in the statistical sample since the struts visible through circular cutting holes in the bubbles cannot be mistaken for walls. The three bubbles forming a strut are the cut bubble and two other ones on each side of a strut, as shown in Figure 6. The short projections, hardly visible below the dim contours of the cut bubbles, should not be disregarded because a short projection may correspond to (a) a short strut having an angle α of ~0° with the image plane or to (b) a long strut having α ≈ 90° with the image plane [12,13]; therefore, the number of short projections can influence the search for PDFs significantly.

Following a similar methodology as in case of bubbles, united selections of N_sp_ = 250–300 projections, l, of struts’ length, L, on three image planes were formed for each microscopy sample. Struts’ projections were measured, taking into account the circular contours of nodes marked on the images. The biggest dimensions of typical struts’ projections in the printed images were ~7–8 mm; therefore, it was convenient to assume 1 mm in the printed image as a nominal unit (1 mm = 1 unit = 0.0057 mm in nature). Each selection was divided in i = 1, 2, ..., 10 classes, each class of width one unit. Relative frequency for each class was calculated as:

m_i_(l) = n_i_(l)/N_sp_, (7)

where N_sp_ is the number of strut projections in a selection and n_i_ is the number of strut projections in the i-th class. Next, histograms were constructed. The variation series of projections and length were approximated with PDFs in the mode of a power law with an exponential cut-off:

f(l, q, b, α) = Al^q^ exp(−αl^b^); f(l, q, b, α) ≥ 0, (8)

(9) $$A=\;1/\int_{-\infty }^{\infty }f\left(l,\;q,\;b,\;\alpha \right)\mathrm{dl},$$

where parameters q = 1.0 and b = 1.5. The values of parameter α were searched for, ensuring the best fit to the experimental data. The struts’ length distribution was determined according to the mathematical model and methodology given in [12,13] by means of data from light microscopy images, taken in three mutually perpendicular planes. A PC code was completed and functions f(L, q, α, b) were determined in 2–3 iterations. The scale of the printed images (A4 format) 1.0000:0.0057 was taken into account when calculating the natural dimensions. In some cases, a lack of the short projections in the statistical sample made a proper choice of the approximating function (i.e., α) problematic.

**Figure 7:**
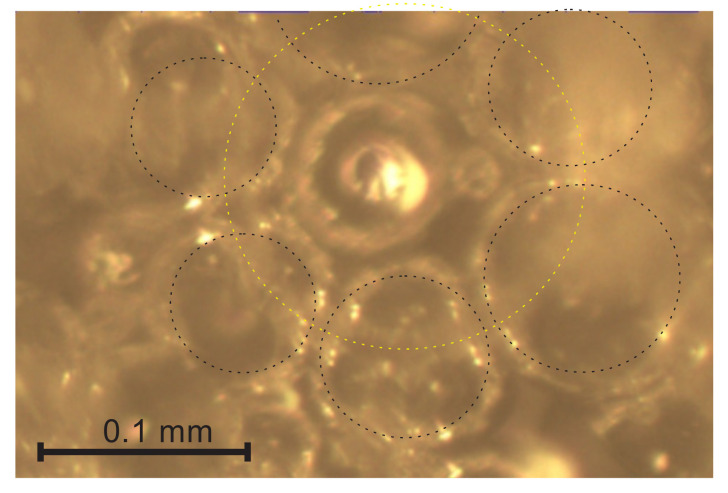
A bubble cut below the equatorial plane.

The short projections may correspond to short struts, as well as to long struts oriented nearly parallel to the vertical axis OX_3_. On the through-cutting surface images, the dim walls of cut bubbles partly cover the short projections. When a bubble (yellow) is cut below its equatorial plane, see Figure 7, the short projections are visible inside and outside the bubble and can be included into the selection. The bubble has contact walls with 6 + 1 = 7 adjacent bubbles (black) below it. When a bubble happens to be cut above the equatorial plane, its walls hinder the visibility of the short projections and only the struts at the bottom of the bubble’s inside are visible, as shown in Figure 6. Therefore, the average values, l_aver_, calculated directly from the experimental data, may differ from the average values calculated from the approximating functions that are constructed, taking into account the well-defined longer projections.

#### 2.4.5. Transversal Dimensions of Struts

The cross section of a strut is a hypocycloid of three cusps [1,4]. The projections of struts’ transversal dimensions, t_sp_, were determined for the same selections of struts as the length projections by measuring a struts’ length projection in the transversal direction from one side to the other, taking into account the scale. It was assumed that the average length of the transversal dimensions’ projections, t_spaver_, differs little from the length of the transversal dimensions, such that t_s_:t_sp_ ≈ t_s_. 

#### 2.4.6. Un-Foamed Volumes

**Figure 8:**
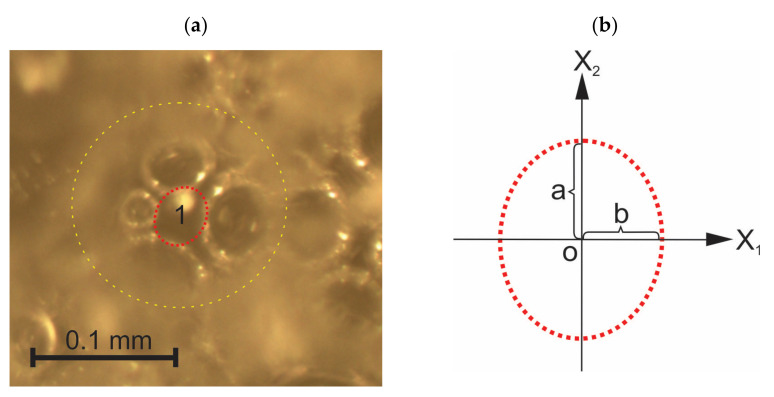
(**a**) An un-foamed volume “1” at the bottom of a cut bubble (yellow) and (**b**) projections “a” and “b” of the semi-axes of the model ellipse (red) on the cutting plane X_1_OX_2_.

Un-foamed polymeric elements of irregular shape that exceed the average dimensions of (a) walls on the cutting surface images or (b) nodes on the through-cutting surface images several times are regarded as un-foamed volumes and are modelled as ellipsoids with semi-axes a, b, and c, as shown in Figure 8.

An elliptic contour was drawn around each un-foamed volume on the printed cutting surface images X_1_ = const., X_2_ = const, and X_3_ = const. Projections of semi-axes were measured, taking into account the scale and the relative area, R_e_ = S_e_/S_0_, occupied by the ellipses on each image of area S_0_ was calculated, where S_e_ is the area of all ellipses on an image. 

### 2.5. XRD Analysis

Intercalation and exfoliation of Cloisite-30B mono-layers was evaluated via the basal spacing by X-ray diffraction (XRD) at a 5 wt.% concentration of nanoclay in dispersions made by (a) high shear mixing, with an effective mixing time of 20 min, and (b) sonication, with an effective sonication time of 20 min. The XRD patterns of clay-polyol dispersions were obtained by a Bruker D8 Discover diffractometer (Bruker AXS GmbH, Karlsruhe, Germany), equipped with a LynxEye detector employed at a 0D mode, using copper radiation (CuKα) at a λ = 0.15418 nm wavelength. The diffractometer’s tube voltage and current were set to 40 kV and 40 mA, respectively. The divergence slit was 0.2 mm, and the anti-scattering slit was 8.0 mm. The samples were spilled into sample holders and rotated during the measurements. XRD patterns were registered at a scan speed of 10 s/0.01° from 1.5° to 7° in the 2θ scale.

## 3. Theoretical

### 3.1. Space-Filling Coefficient 

When a cellular plastic is reinforced with a filler of a certain kind, density and dimensions (short or long fibers, hollow beads, sand, nanoclays, etc.), depending on how the filler is comprised in the foams’ structure, as well as on the technological requirements (e.g., whether the volume or the mass of the foams or both are kept constant etc.), the determination of the space-filling coefficient has to be adapted. The space-filling coefficient, P1, of nanoclay-filled foams, moulded in a sealed mould, is calculated with a requirement that the mass of the blocks (both unfilled and filled), produced in a mould of a constant volume V_0_, is kept constant. Neglecting the mass of gas in the cells, the unfilled monolithic polymer of density ρ_pol_ occupies a volume

V_pol_ = m_pol_/ρ_pol_ = m_0_/ρ_pol_; (10)

where m_0_ is the mass of the reacting mixture. The unfilled monolithic polymer forms the rigid, load-carrying network of walls, nodes, struts, and un-filled volumes. Upon filling, the nanoclay’s particles are fully incorporated in the elements of the network, forming a “Polymer-filler” composite. At the filler’s concentration, η, the mass of filler is calculated as

m_fil_ = ηm_0_. (11)

A filler of density ρ_fil_ occupies a volume

V_fil_ = m_fil_/ρ_fil_ = ηm_0_/ρ_fil_. (12)

To fulfil the requirement m_0_ = const., upon adding a filler, the mass of unfilled polymer forming substances, equal to the mass of monolithic polymer, is reduced for an equal quantity: m_pol_’ = m_0_ − m_fil_. The density of a monolithic polyurethane ρ_0_ ≈ 1280 kg/m^3^ [23] and the density of the Cloisite-30B filler ρ_fil_ ≈ 1980 kg/m^3^ [19], which means that ρ_fil_ > ρ_pol_. At a mass equal to the mass of the added filler, m_fil,_, the un-filled monolithic polymer would occupy a larger volume due to the density difference:

V_pol_’ = m_fil_/ρ_pol_ = ηm_0_/ρ_pol_, V_pol_’ > V_fil_. (13)

A reduction in the volume of the load-carrying network occurs due to filling:

ΔV_poln_ = V_pol_’ − V_fil_ = ηm_0_Δρ/(ρ_pol_ρ_fil_) = ηm_0_(ρ_fil_ − ρ_pol_)/(ρ_pol_ρ_fil_). (14)

The volume, V_polc_, of the “Polymer-filler” composite network:

V_polc_ = V_pol_ − ΔV_poln_. (15)

As such, P1 of filled foams can be calculated as a ratio of volume V_polc_ and volume of foams V_f_.

P1 = V_polc_/V_f_. (16)

Since V_f_ = V_0_, where V_0_ is the inner volume of the mould, the space filling coefficient, P1, and porosity, P2, of filled foams can be calculated in dependence of the clay nanofiller’s concentration:

P1 = (V_pol_ − ΔV_poln_)/V_f_ = m_0_/(ρ_pol_V_0_)[1 − η(ρ_fil_ − ρ_pol_)/ρ_fil_], and (17)

P2 = 1.0 − P1.(18)

### 3.2. Determination of the Bubbles’ Diameters

A mathematical model was described to determine the PDFs of the bubbles’ diameters, f(D), based on PDFs of diameters, d, of bubbles’ cross-sectional circles. Foams are modelled with a number, N_b_, of bubbles of diameters D_1_, D_2_, ..., D_k_ nominal units. The bubbles are arranged into D_k_ classes of width 1 unit. The number of bubbles in a class n_bk_ is determined by the f(D) that is searched for. Two assumptions are made: (1) bubbles of different diameters are distributed randomly in the foam’s material and (2) the experimental data selections of a circle’s diameter is large enough to represent all circles at any cross section.

**Figure 9:**
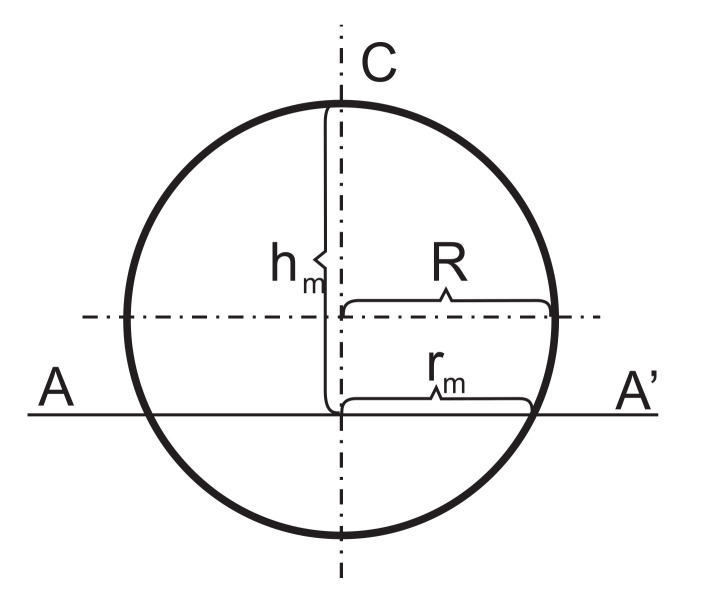
A bubble, with radius R, cut with a plane AA’, where r_m_ is the radius of the m-th circle.

Each bubble is cut with a plane AA’ in M = D/Δh − 1 consecutive positions and M circles of nonzero diameters, d_m_ > 0, are obtained. If h_m_ is the distance of the m-th position from point C (Figure 9), diameter, d_m_, of the m-th circle is calculated as:

(19) $$d_{m}=2r_{m}=2\;\sqrt{r_{m}^{2}-{\left(r_{m}-h_{m}\right)}^{2}},\;\mathrm{where}\;h_{m}=m\Delta h\;\mathrm{and}\;m=1,\;2,\;...,\;M.$$

The number of cutting positions M is equal for each bubble providing M − 1 circles of nonzero diameters d_m_. The calculated values of d are arranged into a matrix; their number, n_ck_, in each class of values is calculated taking into account the number of bubbles providing circles with d values lying in the corresponding class. The relative frequency for each class of circles’ diameters is calculated as:

m_k_(d) = n_ck_/N_c_, (20)

where the total number of circles’ diameters N_c_ = N_b_(M − 1). A histogram of the circles’ diameters, d, is constructed and compared with the experimentally determined one.

The main principle of the model is demonstrated in an example for N_b_ = 20 bubbles of diameters D = 1, 2, ..., 5 units, distributed according to a symmetric f(D), as given in Table 2, where M = 9 cutting positions are considered m = 1, 2, ..., 9, Δh_m_ = D_n_/(M + 1). Five classes are considered: k = 1, 2, …, 5, where k is the ordeal number of a class. Two matrices for numerical calculations of circles’ diameters and their number in each class of values are formed, as given in Table 3 and Table 4.

**Figure 10:**
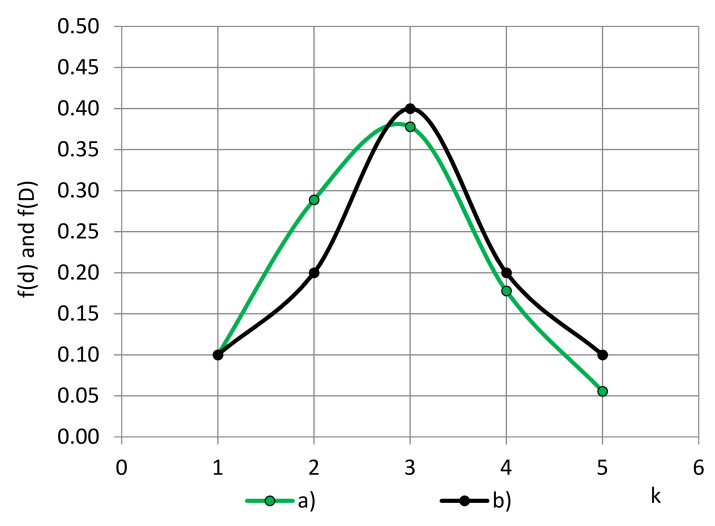
PFDs of diameters of (a) circles—f(d) and (b) bubbles—f(D).

The diameters, d, are counted, grouped into classes, a histogram is constructed, and the approximating function of circles’ diameters, f(d), is determined, as shown in Figure 10.

It is concluded that a symmetric PDF of bubbles’ diameters f(D) provides a slightly asymmetric PDF of diameters of circles f(d). The function f(D) is searched for in the mode of a normal PDF. Varying the parameters of f(D), such a PDF is determined in several iterations, which ensures the function f(d) as a result of the limits of a certain precision. The special cases of the mathematical model are given in the Section S3 of the Supplementary Materials.

The function, f(D), for selections of bubbles’ diameters was searched for in the mode of a normal PDF with parameters μ and σ: f(D, μ, σ). A PC code was compiled and numerical calculations were performed for N1 = 100 bubbles and M = 39 cutting positions. In the first iteration, the function approximating the experimental data of the circles’ diameters was used for generating input data. The function f(d), acquired numerically, was compared to the function approximating the experimental data and a new input function f(D, μ, σ) was constructed for the next iteration, taking into account the trend. Varying μ and σ, f(D, μ, σ) provides the best fitting for the experimental data and was determined in 2–3 iterations. Fitting was evaluated according to Equation (4). The scale, implemented for division in classes (5 mm = 1 unit = 0.0286 mm in nature) was taken into account.

### 3.3. Fraction of Polymer in the Elements

With dimensions of the structural elements known, the fraction of monolithic polymer in polymeric elements-struts, nodes, and walls with typical, average dimensions can be estimated, neglecting the un-foamed volumes whose volume in the given foams is insignificant. If a strut is modelled as a prism of height, L_aver_, and an equilateral triangle, with side t_aver_, at the base, then its volume is:

(21) $$V_{\mathrm{st}}={\mathrm{SL}}_{\mathrm{aver}}=\sqrt{3}/4t_{\mathrm{aver}}^{2}L_{\mathrm{aver}}.$$

The volume of a spherical node of diameter d_aver_ equals V_n_ = 3/2π(L_aver_)^3^. A wall is modelled as a round cylinder with spherically concave bases of equal curvature (For simplicity), height t_wav_^p^, and volume V_w_:

V_w_ = πt_wav_^p^ (d_waver_)^2^ – 1/3πh_segm_ [(h_segm_)^2^ + 3r^2^], (22)

where h_segm_ = (t_waver_^p^ − t_waver_^c^)/2 and r = d_waver_/2 are the height and the base radius of the two spherical segments. Assuming that the number of walls, struts, and nodes in PU foams’ material is approximately equal, the total amount of polymer in a unit volume element of PU foams is expressed as:

φ_w_ + φ_st,n_ = 1.0, φ_w_ = V_w_/(V_w_ + V_st_ + V_n_) and φ_st,n_ = V_st,n_/(V_w_ + V_st_ + V_n_), (23)

where φ_w_ is the the fraction of polymer comprised in walls and φ_st,n_ is the same in struts and nodes. 

## 4. Results and Discussion

### 4.1. Characteristics of the Light Microscopy Samples

The density of the microscopy samples lies in the limits between ~210–230 kg/m^3^, as detailed in Table 5. Assuming that the whole block of volume V_0_ comprises PU foams of density equal to that of a microscopy sample, the mass, m, for each block is calculated. Values of m_1_, m_2_, ..., m_7_ permit one to calculate the space-filling coefficient, P1, and porosity, P2, of each microscopy sample. It can be seen that the density differences of the samples are less than 10%. The porosity values are similar and lie in the limits of 83.5–83.8%. The highest porosity is identified at a filler concentration η = 5%. Characteristics of the moulded PU foams’ blocks are given in Section S4 of the Supplementary Materials.

### 4.2. Characteristics of Structural Elements

#### 4.2.1. Bubbles

An analysis of the images in three planes X_1_ = const., X_2_ = const., and X_3_ = const. of each block showed that nearly all cross-sections of the bubbles are circular, and there were no polyhedral shapes. On images X_1_ = const. and X_2_ = const., slightly elliptical contours were observed (ratio of semi-axes 1.03–1.10) that were modelled as circles with a diameter equal to the average of the ellipse’s semi-axes. Next, from geometrical considerations, it can be assumed that the shape of the bubbles is spherical and the structure of the foams is isotropic; therefore, the three selections from a microscopy sample were united into a single selection with N_c_ = 200–250 circles.

**Figure 11:**
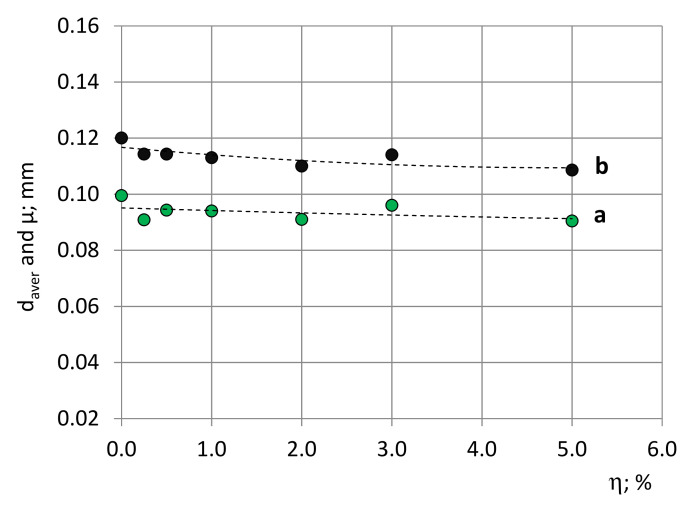
Average diameters of (a) circles d_aver_ (experimental data) and (b) bubbles D_aver_ = µ (PDFs).

By means of the developed mathematical model (Section 3.2), the distribution characteristics of the bubbles’ diameters were determined, as given in Table 6 and Figure 11. The distribution characteristics of the circles’ diameters are given in Section S5 of the Supplementary Materials.

It can be seen from Table 6 that the relative differences in the average diameters of the bubbles are ≤10%. The relative increase in the number of cells due to the filler’s concentration is 20–40%. The biggest number of bubbles per unit volume corresponds to the highest concentration of nanoclay, being 5%, since the filler particles act as a nucleating agent in the foaming process. The calculated values of N_b_ are in appropriate correspondence with those given in [18,19] for rigid PU foams of a density of ~200 kg/m^3^.

Figure 11 gives the average diameters, d_aver_, of the circles as determined experimentally, as well as the bubbles’ average diameters, D_aver_. The dimensions of the bubbles are the smallest at the highest filler concentration of 5%. The calculated values of the bubbles’ average diameters, μ, are in appropriate logical correspondence with previously reported results [15,18,19]. In [15], for high-density PU foams, ρ_f_ = 389 kg/m^3^, the average actual, restored diameter of the bubbles is reported as µ ≈ 104 µm, and in [18,19], for Neopolyol-380 PU foams, ρ_f_ ≈ 200 kg/m^3^ and a Cloisite-30B filler of 0.0–1.5%, the SEM measured average diameters of cutting circles that were determined as 140–190 µm.

**Figure 12:**
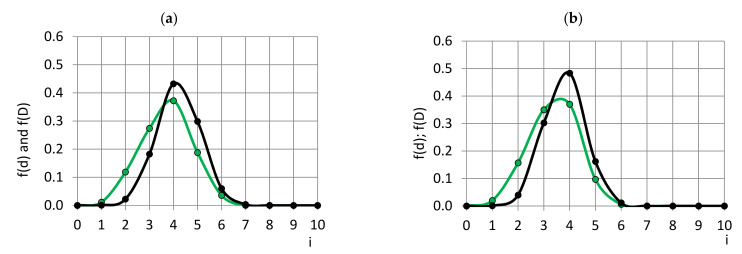
PDFs of circles’ diameters, d, (green) and bubbles’ diameters, D, (black) of PU foams; concentration of filler (**a**) η = 0.0% and (**b**) η = 5.0%.

The probability density functions, f(d) and f(D), of the circles’ and bubbles’ diameters, respectively, for neat, η = 0%, and, as a comparison, for filled, η = 5%, PU foams are given in Figure 12. It can be seen that the filler has narrowed the range of the bubbles’ variation in diameter—the 5% nanoclay-filled foams have a more uniform cellular structure than neat foams. At η = 5%, the maximum of the bubbles’ diameters has shifted to the smaller side of values, meaning that in the filled foams, the cells are smaller. The PDFs lack elements in the class №1 (Range 0.0–0.029 mm), suggesting that practically all bubbles have grown at least above the size of a diameter of ~0.029 mm.

#### 4.2.2. Walls

**Figure 13:**
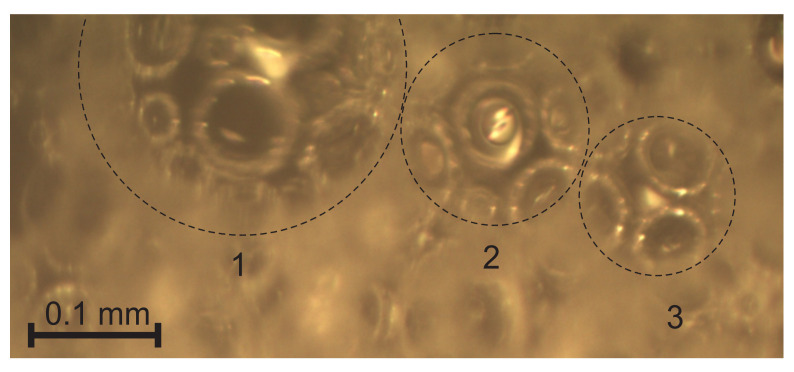
Front view of the walls (visible through the large cut bubbles 1, 2, and 3 between a large bubble and a number of smaller ones.

In contrast to low-density foams, in the foams of ρ_f_ ≈ 210–230 kg/m^3^, the diameters of the bubbles differ greatly and a bubble of comparatively large diameter can have an interface with a number of smaller ones, as illustrated in Figure 13. Neither the bubbles nor the walls are polyhedral. No open cells were identified, which corresponds with results of water absorption tests that identified ~99% closed-cell structure [19]. On the through-cutting surface images, the front view of nearly all walls is circular and only the walls located at an angle to the image plane are imaged as elliptical. It is further assumed that all the walls are circular.

**Figure 14:**
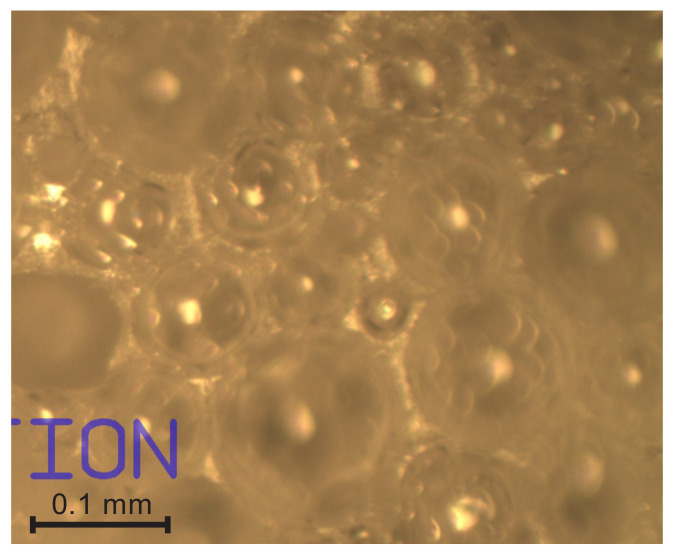
Walls between bubbles of similar dimensions.

The walls’ cross sections are biconcave, with the different curvatures of each side depending on the diameters of the contacting bubbles. The thickness, t_w_, is the smallest in the centre and increases in the perimetral direction, as shown in Figure 5. In some locations, a number of bubbles has similar dimensions, and, possibly due to pressure balance, the structure features a hexagonal close order, as illustrated in Figure 14.

The wall’s average diameter, d_waver_, and average thickness in the centre, t_waver_^c^, as well as along the perimeter, t_waver_^p^, measured from printed images of three cutting surfaces, are given in Table 7 for selections of 100–150 typical walls, for each of the seven microscopy samples; coefficients of variation range from 35 to 40%. The average diameter of walls decreases with an increase of the concentration of filler for ≈15%. The smallest average diameter corresponds to the highest filler concentration of 5%; therefore, the walls between the bubbles of the 7-th block foams have the most curved surface. The ratio of the average perimetral thickness and the average thickness in the wall’s centre, ξ_1_ = t_waver_^p^/t_waver_^c^, changes within the limits of 1.20 to 1.70. 

In [24], the experimentally determined cell face thickness values for PU foams, ρ_f_ ≈ 100 kg/m^3^, are reported as 0.004–0.008 mm, which displays a proper trend for PU foams of nearly two times smaller density.

#### 4.2.3. Nodes

The smallest nodes were identified in the PU foams sample with a filler concentration of 5%. The values of the ratio ξ_2_ = d_naver_/t_aver_ show that the nodes’ average diameter, d_naver_, exceeds the average transversal dimension, t_aver_, of struts by 1.6–1.7 times, as given in Table 8, meaning that the nodes are properly distinguished from the un-foamed volumes. It can be seen that, although the absolute values of d_naver_ and t_aver_ change with an increase in the filler concentration, the ratio ξ_2_ remains relatively constant and exhibits no definite trend. 

#### 4.2.4. Length of Struts

**Figure 15:**
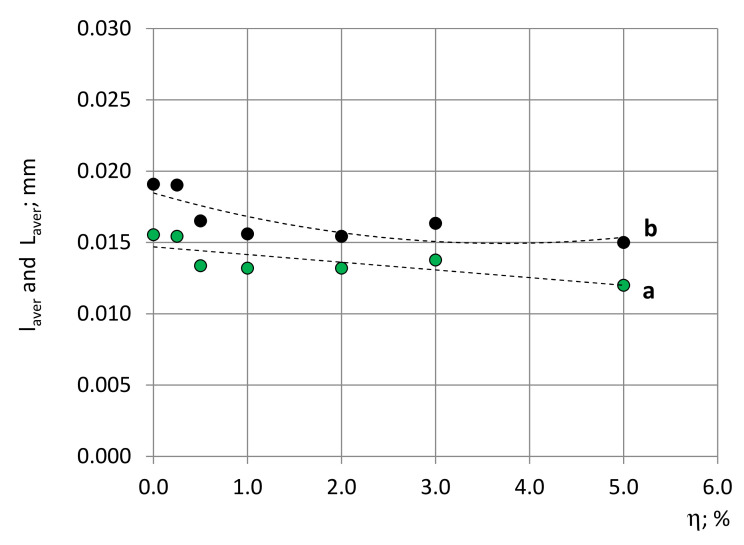
(a) Struts’ average length projections, l_aver_ (experimental data, approximating functions), and (b) Average length, L_aver_ (approximating functions), in dependence on the nanoclay concentration, η.

The struts’ average length projections, l_aver_ (experimental data), and average length, L_aver_, determined according to the approximating functions, are given in Figure 15. The average values, l_aver_, calculated directly from the experimental data, for the same blocks differ from the average values calculated from the approximating functions of the histograms. The differences are caused by the problems described in Section 2.4.4 to identify the short projections of struts on the images that lead to shortage of short projections in the experimental data histograms. Therefore, the approximating functions of histograms are constructed, taking into account the properly determined number of longer projections. The distribution characteristics of the struts’ length projections and length are given in the Section S6 of the Supplementary Materials.

The relative difference between the struts’ average projection’s length and the struts’ average length is ~20–25%, which is in a good agreement with the conclusions of [13] for isotropic PU foams. Since the medium-density foams considered in this study are isotropic, the angular distribution of struts, as characterised by a certain spatial angle, dω, corresponding to each strut, is even. The relative differences in the struts’ average length, L_aver_, at different concentrations of filler are ≤20%, meaning that large selections from different images are necessary to identify the main trends. With an increase in the concentration of filler, the struts gradually become shorter, as evidence in Figure 15. It can be seen that, with a decrease in the size of bubbles, the average length of struts decreases as well, meaning that the number of struts per unit volume increases. The shortest are the struts in the block №7, with a filler concentration of η = 5%, where the biggest number of bubbles in a unit volume was identified.

#### 4.2.5. Transversal Dimensions of Struts

The average transversal dimensions of struts, t_saver_, are given in Table 9. The biggest values of t_saver_ were identified in the neat PU foams sample: t_saver_ ≈ 0.020 mm. For the filled foams, the average transversal dimensions were quite similar: 0.017–0.018 mm. The struts’ slenderness ratio was introduced as ξ_3_ = L_aver_/t_saver_, where L_aver_ is the average length of the struts. It can be seen that the transversal dimensions are similar or larger than the longitudinal, where ξ_3_ ≤ 1.0, which means the in the density range, the struts are not slender. The PU foams considered here feature a transition from a strut-like structure to that of isolated bubbles in a polymeric matrix where struts already can’t be distinguished as structural elements.

#### 4.2.6. Un-Foamed Volumes

Assuming that the projections of the semi-axes’ of the model ellipsoid of an un-foamed volume differ little from the semi-axes themselves, a_p_ ≈ a, b_p_ ≈ b, and c_p_ ≈ c, it was estimated that the range of the natural dimensions of semi-axes a, b, and c is practically the same for all seven blocks of PU foams, where 0.05 mm < a, b, and c < 0.15 mm and R_e_ = 3 ... 5% on all images. That corresponds to the relative amount of un-foamed volumes in a volume element of 0.5 to 1.0%. The part of the monolithic polymer that can be considered as an un-foamed volume is very small at all concentrations of filler. The nucleation of bubbles has been quite uniform in the volume of the produced PU foams. 

### 4.3. Fraction of Polymer in Structural Elements

According to numerical calculations that used the experimentally determined dimensions of the medium-density, neat PU foams’ structural elements as input data, the volume of polymer in a wall of PU foams exceeds the summary volume of a node and a strut by 2–3 times: V_w_/(V_st_ + V_n_) ≈ 2.5. Next, the corresponding volume fractions can be estimated as 66% ≤ φ_w_ ≤ 75% and 34% ≥ φ_st,n_ ≥ 25%. In contrast to low-density PU foams, where >95% of polymer is concentrated in the struts and nodes, and the walls are extremely thin [1,2,3,4], the load bearing capacity of the relatively massive walls should be considered along with that of struts and nodes.

### 4.4. Results of the XRD Analysis

Similar XRD-patterns were acquired for the 5 wt.% Cloisite-30B dispersions made by (a) high shear mixing for 20 min and (b) sonication for 20 min. The characteristic changes in the diffraction patterns were observed: the displacement of the angular position of the 001 reflex to the smaller angles, which is a consequence of the penetration of macro-chains into the galleries, and a decrease in the intensity of the diffraction peak, due to the mechanical delamination of clay particles under the action of shear forces.

**Figure 16:**
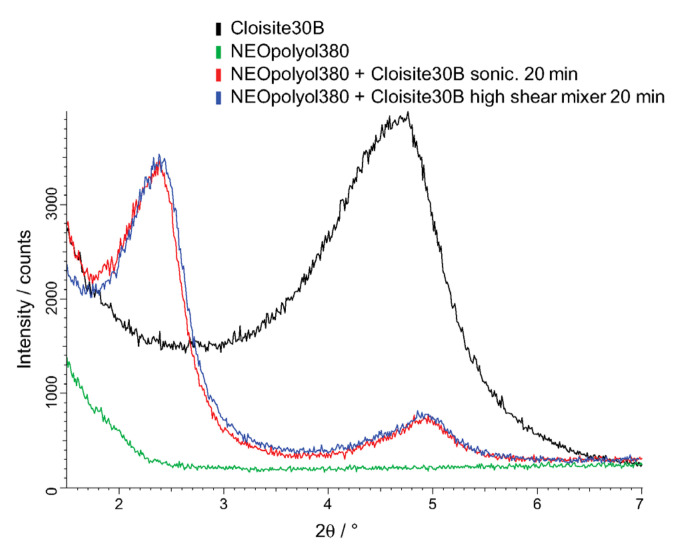
XRD-patterns of (**black**) Cloisite-30B, (**green**) NEOpolyol-380, or a 5% dispersion of Cloisite-30B in NEOpolyol-380 made by (**red**) sonication for 20 min or (**blue**) high shear mixing for 20 min.

The diffraction peaks have shifted to the left compared to that of pure Cloisite-30B, indicating a change in d-spacing between diffraction lattice planes according to Bragg’s law, d = nλ/(2sinθ), as illustrated in Figure 16. The first diffraction peak (n = 1) has shifted from 2θ = 4.75° to 2.38°, which corresponds to an increase of d-spacing from 18.6 Å to 37.1 Å. The second diffraction peak (n = 2) is identified at 2θ ≈ 4.95°, which corresponds to a d-spacing of 35.7 Å. During subsequent polymerization, the polymer chains grow, facilitating the expansion of interlayer spacing and the exfoliation of nanoclay platelets in the polyurethane matrix. The nanoclay has not fully exfoliated since the diffraction signals are still visible and intercalation is dominating. 

## 5. Conclusions

A methodology is developed for light microscopy investigation of the complex structure of rigid, medium-density, closed-cell PU foams, with a density of 210 kg/m^3^–230 kg/m^3^, and a porosity of 83.5–83.8%, by taking two kinds of images of one and the same location on a sample’s surface. The method is appropriate to gather sufficiently large selections of circles, struts’ projections, and projections of other elements. The identification of the short projections of struts on the through cutting surface images turned out to be a challenge because the cut bubbles hinder visibility. Therefore, the approximating functions of histograms have to be determined, taking into account the more clearly visible longer projections.

The estimate of polymer distribution between the structural elements showed that, in contrast to low-density PU foams, where >95% of polymer is concentrated in struts and nodes and the walls are extremely thin, in medium-density PU foams, the relatively massive walls have be taken into account along with the struts, e.g., when estimating the load-bearing capacity of the structural elements.

The described mathematical model permitted the determination of the actual dimensions and probability density functions of the bubbles’ diameters. The average dimensions of the spherical bubbles at all filler concentrations are ~5–6 times bigger than the average length of the struts. The bubbles are situated relatively far from each other. In light-weight PU foams, with a density of 40–50 kg/m^3^, having an expressed strut-like structure, the average diameter of the bubbles is only ~1.1–2.0 times bigger than the average length of the struts, and the bubbles are located close to each other and have a polyhedral shape. Taking into account the fact that the struts are not slender (slenderness ratio ≤ 1.0), it can be concluded that the medium-density PU foams considered here feature a transition from a strut–like structure to that of isolated, gaseous bubbles in a polymeric matrix.

Light microscopy showed that the PU foams that are moulded in a sealed mould are nearly isotropic and that using the nanoclay Cloisite-30B, up to a 5% concentration, as a filler has not changed the isotropic character of the cellular structure. With an increase of the nanofiller’s concentration from 0 to 5%, the average diameter of the bubbles decreased gradually by ~10% and the average length of struts decreased by ~10–20%. In turn, the average number of bubbles per unit volume increased by ~20–40% and reached the biggest value of 1.24 × 10^6^/cm^3^ at the highest concentration of nanoclay, 5%, since the filler’s particles act as bubbles’ nucleation sites.

The acquired data might be useful in mathematical models of “Structure-mechanical properties” based on (a) model-cells of different geometry and (b) a singular structural element, such as “Strut-node”, etc., as well as for the comparison and verification of SEM- and X-ray-computed tomography data. Further research might deal with light microscopy of free rise, medium-density PU foams, where structural anisotropy can be expected.

## Figures and Tables

**Figure 1:**
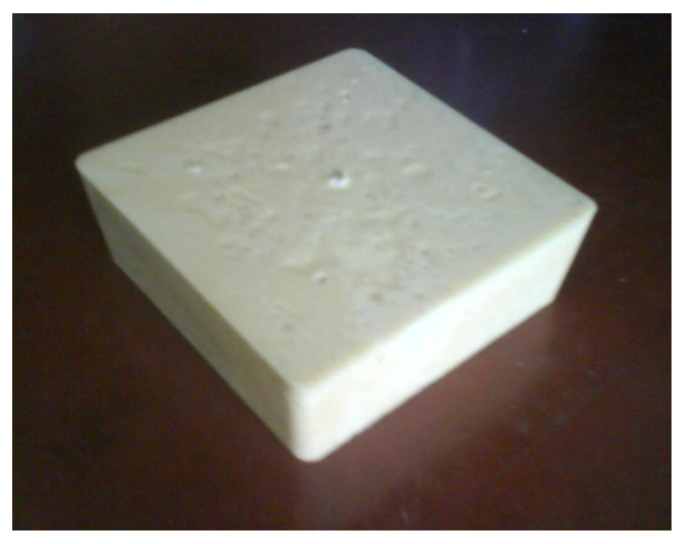
A rigid PU foams’ block, molded in a sealed steel mould.

**Table 1:** The formulation of the PU foams.

Polyol Formulation; pbw *		
1	Recycled APP NEOpolyol-380	80.0
2	Cross-linking agent, Lupranol 3422	20.0
3	Flame retardant, TCPP	20.0
4	Blowing agent, water	1.0
5	Reactive catalyst, PC CAT NP 10	1.6
6	Surfactant, NIAX Silicone L6915	2.0
Polyisocyanate; pbw		193
**Formulation characteristics**		
1	Recycled materials in PU foams, %	15
2	Isocyanate index	160
**Technological parameters**		
1	Cream time, s	25
2	String time, s	45
3	Tack free time, s	60
4	Foaming end time, s	60

* Parts by weight per hundred parts of polyol.

**Table 2:** Distribution of bubbles’ diameters (an example).

Bubbles’ Diameters D, Units	Function f(D)	Number in Class n_bk_
1	0.1	2
2	0.2	4
3	0.4	8
4	0.2	4
5	0.1	2
Sum Σ	1.0	20

**Table 3:** A matrix for the numerical calculation of circles’ diameters.

Class	0 ≤ D ≤ 1	1 < D ≤ 2	2 < D ≤ 3	3 < D ≤ 4	4 < D ≤ 5
D; units	1.0	2.0	3.0	4.0	5.0
R; units	0.5	1.0	1.5	2.0	2.5
n_bk_	2	4	8	4	2
k	Circles’ diameters d = 2r				
1	0.60	1.20	1.80	2.40	3.00
2	0.80	1.60	2.40	3.20	4.00
3	0.92	1.83	2.75	3.67	4.58
4	0.98	1.96	2.94	3.92	4.90
5 (Equatorial)	1.00	2.00	3.00	4.00	5.00
6	0.98	1.96	2.94	3.92	4.90
7	0.92	1.83	2.75	3.67	4.58
8	0.80	1.60	2.40	3.20	4.00
9	0.60	1.20	1.80	2.40	3.00

**Table 4:** A matrix for the numerical calculations of a number of circles’ diameters, d, in a class.

Class	0 ≤ d ≤ 1	1 < d ≤ 2	2 < d ≤ 3	3 < d ≤ 4	4 < d ≤ 5	Sum Σ
**k**	**n_c_**					
1	2	12	6	0	0	20
2	2	4	8	6	0	20
3	2	4	8	4	2	20
4	2	4	8	4	2	20
Sum Σ_1_	8	24	30	14	4	80
Sum Σ_1_ × 2	16	48	60	28	8	160
5 (Equatorial plane)	2	4	8	4	2	20
Sum Σ_2_	18	52	68	32	10	180
m_k_ (d)	0.10	0.29	0.38	0.18	0.06	1.00

**Table 5:** Characteristics of light microscopy samples.

Concentration η; %	Density ρ_f_; kg/m^3^	Space Filling Coefficient P1; %	Porosity P2; %
0.00	211	16.5	83.5
0.25	220	16.4	83.6
0.50	219	16.4	83.6
1.00	224	16.4	83.6
2.00	232	16.3	83.7
3.00	217	16.3	83.7
5.00	216	16.2	83.8

**Table 6:** Distribution characteristics of the bubbles’ diameters.

Concentration η; %	Average Diameter μ; mm	Standard Deviation σ; mm	Coefficient of Variation v; %	Number of Bubbles N_b_; cm^−3^
0.00	0.120	0.023	19	0.89 × 10^6^
0.25	0.114	0.023	20	1.05 × 10^6^
0.50	0.114	0.023	20	1.08 × 10^6^
1.00	0.113	0.029	25	0.89 × 10^6^
2.00	0.110	0.020	18	0.99 × 10^6^
3.00	0.114	0.020	18	1.04 × 10^6^
5.00	0.109	0.020	18	1.24 × 10^6^

**Table 7:** Characteristics of the walls’ dimensions.

Concentration η; %	Average Diameter d_waver_; mm	Average Thickness (Center) t_waver_^c^; mm	Average Thickness (Perimeter) t_waver_^p^; mm	Ratio ξ_1_
0.00	0.051	0.014	0.021	1.47
0.25	0.049	0.014	0.020	1.43
0.50	0.047	0.012	0.020	1.67
1.00	0.049	0.011	0.017	1.55
2.00	0.048	0.013	0.016	1.23
3.00	0.047	0.011	0.015	1.36
5.00	0.043	0.011	0.016	1.45

**Table 8:** Characteristics of the nodes’ diameters.

Concentration η; %	Average Diameter d_naver_; mm	Standard Deviation s; mm	Coefficient of Variation v; %	Ratio ξ_2_
0.00	0.031	0.008	25	1.56
0.25	0.028	0.005	16	1.63
0.50	0.028	0.005	19	1.68
1.00	0.030	0.005	16	1.66
2.00	0.029	0.005	17	1.60
3.00	0.027	0.005	18	1.58
5.00	0.026	0.006	21	1.54

**Table 9:** Characteristics of the struts’ transversal dimensions.

Concentration η; %	Average Transversal Dimension t_saver_; mm	Standard Deviation s; mm	Coefficient of Variation v; %	Slenderness Ratio ξ_3_
0.00	0.020	0.005	23	0.94
0.25	0.018	0.004	20	1.06
0.50	0.017	0.004	26	1.01
1.00	0.018	0.004	20	0.88
2.00	0.018	0.004	20	0.82
3.00	0.017	0.003	21	0.95
5.00	0.017	0.003	20	0.88

## Data Availability

Data is contained within this article.

## Supplementary Materials

The following supporting information can be downloaded at: https://www.mdpi.com/article/10.3390/polym14061154/s1, Figure S1. A scheme of PU foams’ production. Figure S2. Production of a rigid PU foams’ block: (a) An open mould, (b) A mould with a sealed lid and (c) A mould with a PU foams’ block. Figure S3. Depth of field imaged on a micrograph of 4 razor blades. Figure S4. Rigid, low density, free-rise, closed-cell PU foams; ρ_f_ = 45 kg/m^3^ (P1 = 4%), image plane: (a) Perpendicular to the rise direction OX_3_ and (b) Parallel to the rise direction. Figure S5. Histograms for the special cases of the mathematical model. Figure S6. Space filling coefficient P1 of the moulded blocks in dependence of filler’s concentration η: Theoretical estimation (black) and Experimental data (green). The dashed lines-the corresponding trendlines. Figure S7. Histograms of circles’ diameters of PU foams (a) η = 0.0% and (b) η = 5.0%. Figure S8. Histograms of struts’ length projections of PU foams (a) η = 0.0% and (b) η = 5.0%. Figure S9. PFD-s of struts’ length projections l (Green) and length L (Black) of PU foams; concentration of filler (a) η = 0.0%; and (b) η = 5.0%. Table S1. Characteristics of the produced PU foams’ blocks (Experimental data). Table S2. Distribution characteristics of circles’ diameters. Table S3. Distribution characteristics of struts’ length projections l and length L.
