# Supplementary material for: Light Microscopy of Medium-Density Rigid Polyurethane Foams Filled with Nanoclay
Source: Polymers (Basel). 2022 Mar 14;14(6):1154. doi: 10.3390/polym14061154 (PMC8955111; doi:10.3390/polym14061154)
Supplement: Supplementary file 1 [file polymers-14-01154-s001.zip › polymers-1622626-supplementary.pdf]

polymers

MDPI

Light Microscopy of Medium-Density Rigid Polyurethane Foams Filled with Nanoclay

Ilze Beverte, Ugis Cabulis, Janis Andersons, Mikelis Kirpluks, Vilis Skruls and Peteris Cabulis

1. PU foams' production in a sealed mould

The polyol component was made by weighing the components (The recycled polyol NEOpolyol-380, cross-linking agent Lupranol 3422, flame retardant, blowing agent, catalyst and surfactant) and stirring them for 1 min with a mechanical stirrer at 2000 rpm, Figure S1.

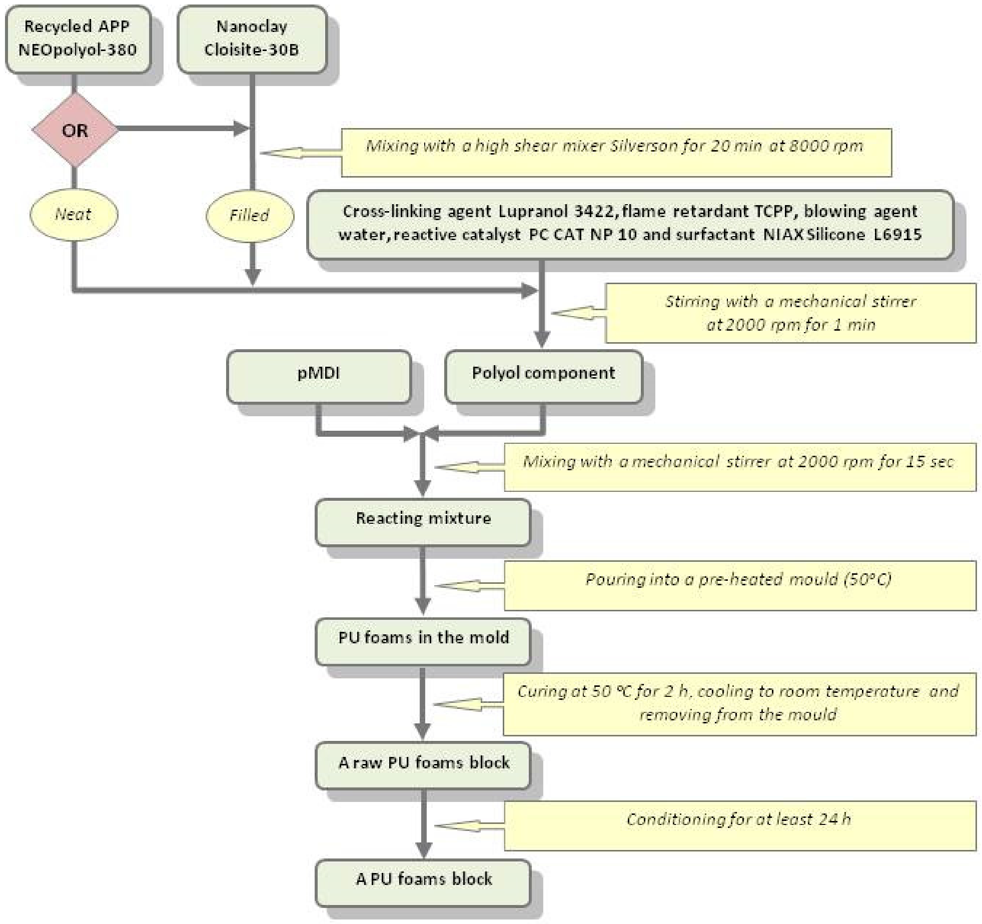

Figure S1. A scheme of PU foams' production.

The pMDI and polyol components were weighted and mixed by a mechanical stirrer at 2000 rpm for 15 sec. An appropriate amount of the reacting mixture was poured into the mould that was preheated to 50°C and the mould was sealed, Figure S2. Mass of the reacting mixture was calculated as to obtain PU foams with an approximate desired apparent density.

(**a**) (**b**) (**c**)

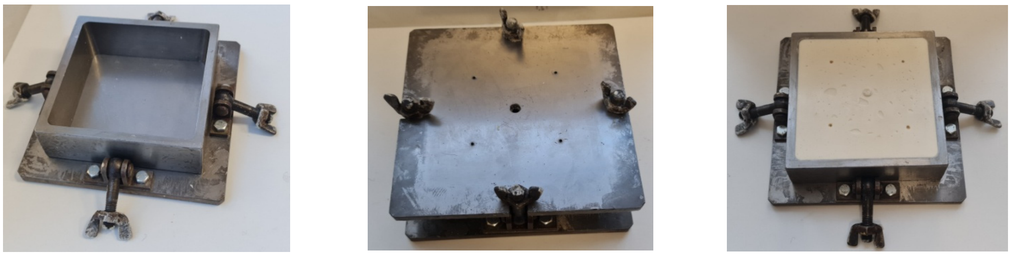

Figure S2. Production of a rigid PU foams' block: (**a**) An open mould, (**b**) A mould with a sealed lid (**c**) A mould with a PU foams' block.

The mixed-in air escaped from the mould through a gas-release hole, which was sealed after all air had escaped. Then the PU foams were cured at 50 °C for 2 h. The mould was cooled to the room temperature; PU foams' block was removed and conditioned for at least 24 h.

2. Depth of field and comparison to low density PU foams

To investigate how the light microscope's limited depth of field displays itself on the micrographs, 4 razor blades, each of thickness 0.10 mm, were pressed together firmly, with a slight offset with respect to each other at their short, dull edges. At the overlapping the surfaces of blades form a step-like structure with a known height difference (0.10 mm). Then the focus was adjusted on the surface of the 2-nd blade and an image was taken at magnification 10 X, Figure S3. It can be seen that the surfaces 1, 3 and 4, situated at known heights (0.10 mm lower, 0.10 mm higher and 0.20 mm higher) with respect to the focused surface 2 (Imaged sharply), are imaged at different grades of dimness (Not sharply). That information was useful for estimation of the mutual spatial disposition of PU foams' structural elements (Struts, nodes, walls etc.), imaged on the micrographs.

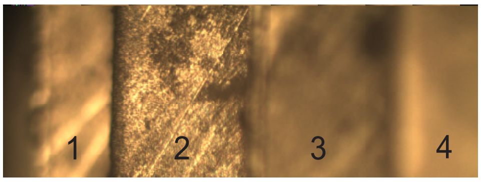

Figure S3. Depth of field imaged on a micrograph of 4 razor blades.

As a comparison to the investigated medium density PU foams, light microscopy images of low density rigid PU foams, ρ_f < 100 - 150 kg/m^3, P1 < 10 % are given in Figure S4. These foams, applied in thermal insulation, have a pronounced strut-like structure with slender struts of nearly constant cross-section and small, regular nodes [1]. The low density PU foams are often produced in free-rise, therefore a considerable structural anisotropy may exist. Only a small amount of monolithic polymer is concentrated in cells' walls. The walls are polyhedral, flat, and comparatively thin and are not regarded as load-carrying elements. The thin walls are sufficiently transparent and cause no interference on the through-cutting images. Large areas of sharply imaged struts and nodes are visible through the cutting surface. That makes the light microscopy of low density PU foams advantageous in comparison to that of medium density ones.

(**a**) (**b**)

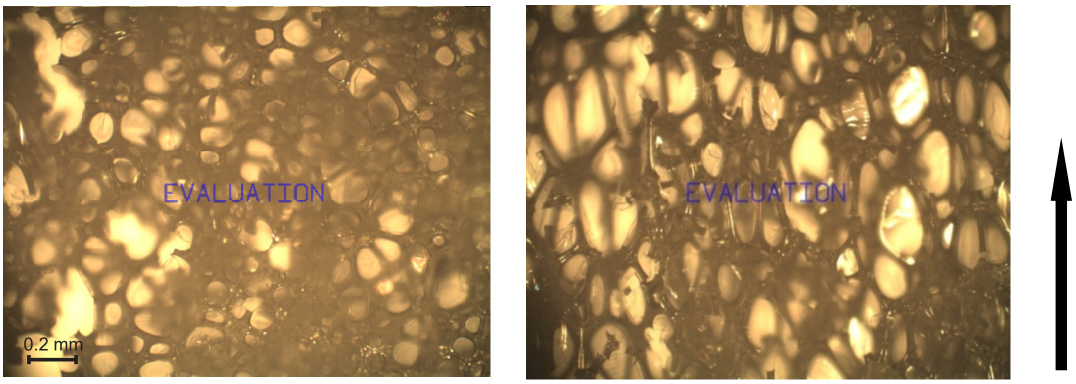

OX3

Figure S4. Rigid, low density, free-rise, closed-cell PU foams; ρ_f = 45 kg/m^3 (P1 = 4 %), image plane: (**a**) Perpendicular to the rise direction OX_3 and (**b**) Parallel to the rise direction.

3. Special cases of the mathematical model

Several special cases are analyzed to test the model: 1) All bubbles have an equal diameter D = 10 units, 2) Diameters D = 1, 2, ..., 10 units have equal probability f(D), diameters D are distributed according to 3) A triangular, increasing PDF, 4) A triangular, decreasing PDF and 5) A normal PDF. The results, Figure S5, are in a good correspondence with results in [2,3].

1.1)

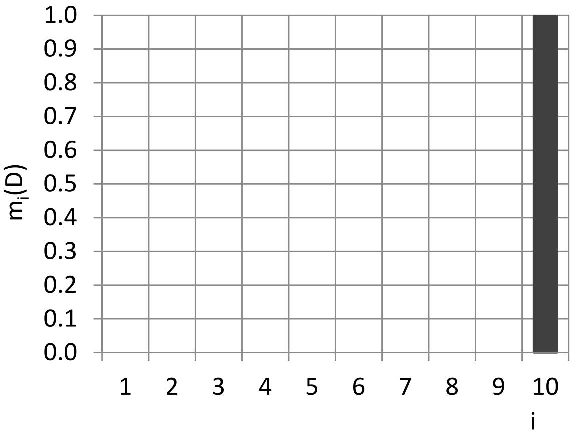

1.2)

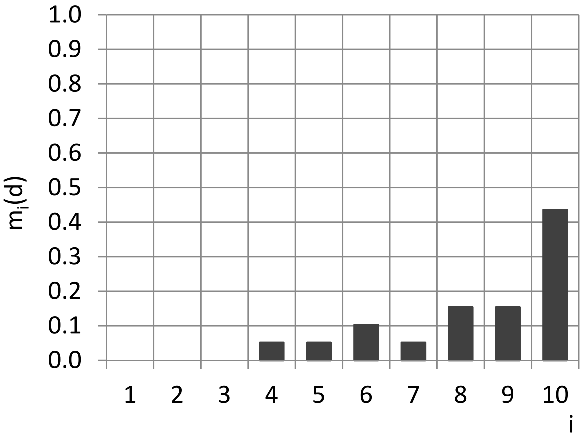

2.1)

2.2)

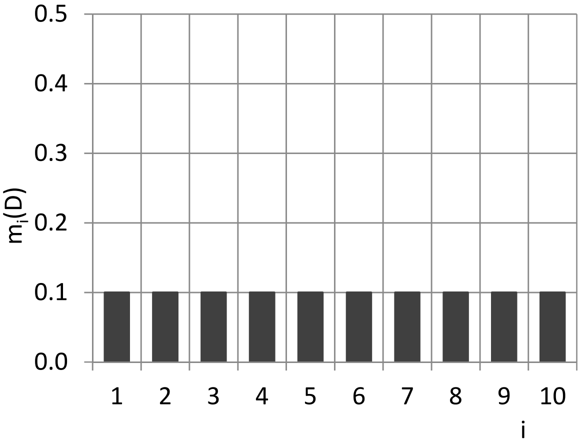

3.1)

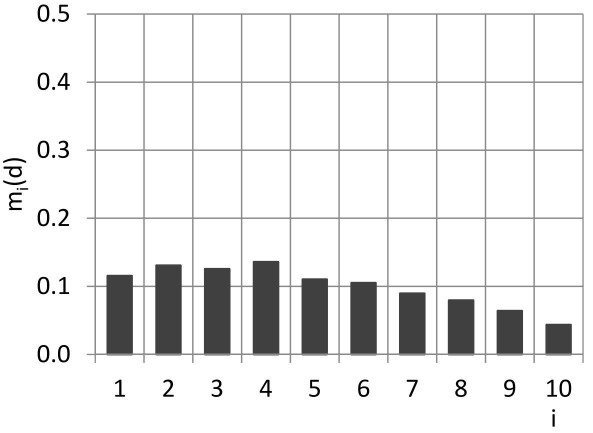

3.2)

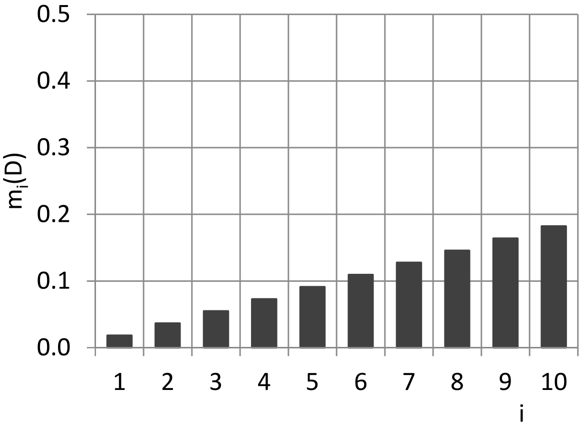

4.1)

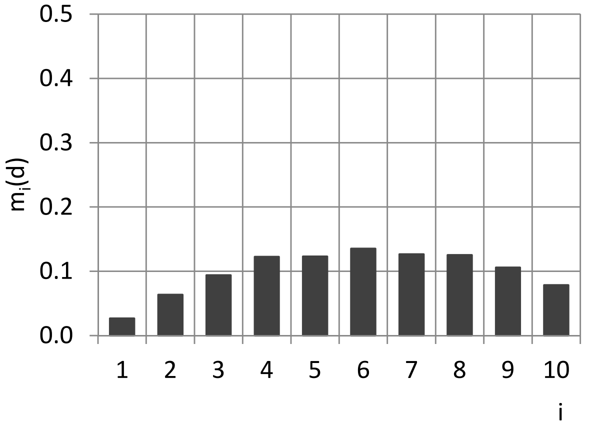

4.2)

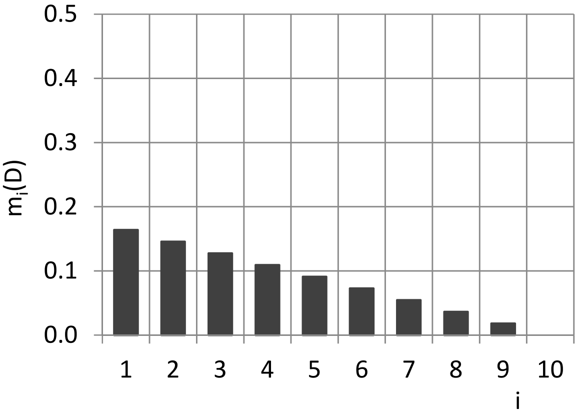

5.1)

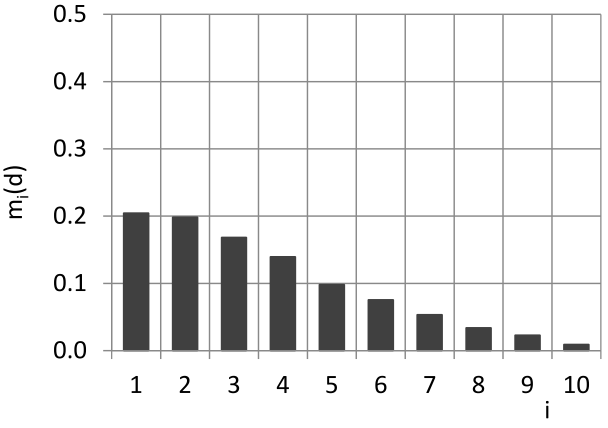

5.2)

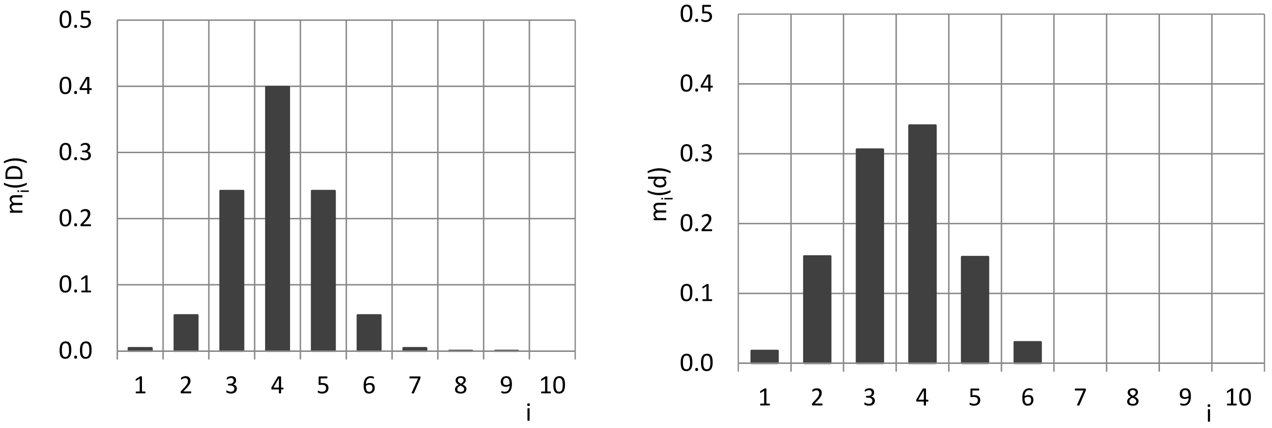

Figure S5. Histograms for the special cases of the mathematical model.

4. Characteristics of the moulded PU foams' blocks

Seven rigid PU foams' blocks of apparent overall density $\varrho_{fa} = \rho_{fa} = m_n/V_0 = 232 \ldots 248$ kg/m^3 (Skins included), were produced, Table S1, where m_n is actual value of mass of the n-th block, n = 1, 2, …, 7. The average content of closed cells of neat PU foams was determined as 99 %. Density in free rise of neat PU foams of the given formulation was determined as 145 kg/m^3. The relative difference R_{mn} between the mass of the neat foams' block, n = 1, and that of the n-th filled block was calculated:

$$R_{mn} = |\Delta m_n/m_1|; \text{ where } \Delta m_n = m_n - m_1 \text{ and } n = 2, ..., 7. \quad \text{(S1)}$$

For the apparent overall density the relative difference R_d:

$$R_{dn} = |\Delta\varrho_{fan}/\varrho_{fa1}| \leq 4 \text{ \%}; \text{ where } \Delta\varrho_{fan} = \varrho_{fan} - \varrho_{fa1}. \quad \text{(S2)}$$

It can be seen that the relative differences R_{mn} and R_{dn} don't exceed 4%, therefore the technological requirement m_0 = const. can be considered as properly executed.

Table S1. Characteristics of the produced PU foams' blocks (Experimental data).

Concentration η; %	Mass m_n; g	Mass diference Δm_n; g	Relative mass difference R_{mn}; %	Density ϱ_{fan}; kg/m^3	Density difference $\Delta\varrho_{fan}$; kg/m^3	Relative density difference R_{dn}; %	Space filling coeffic. P1; %	Porosity P2; %
0.00	250	0	0	238	0	0	18.6	81.4
0.25	253	3	1	240	-3	1	18.8	81.2
0.50	245	-5	2	233	5	2	18.2	81.8
1.00	249	-1	0	237	1	0	18.5	81.5
2.00	261	11	4	248	-11	4	19.3	80.7
3.00	244	-6	2	232	5	2	18.0	82.0
5.00	258	8	3	245	-7	3	18.8	81.2

Assuming density of neat monolithic polyurethane $\varrho_0 \approx 1280$ kg/m^3 [4] and density of the Cloisite-30B filler $\varrho_{fil} \approx 1980$ kg/m^3 [5], the theoretical dependence of space filling coefficient P1 on the concentration of filler η was calculated (Point 3.1 of the article) and depicted in Figure S6 together with P1 values of the seven moulded blocks.

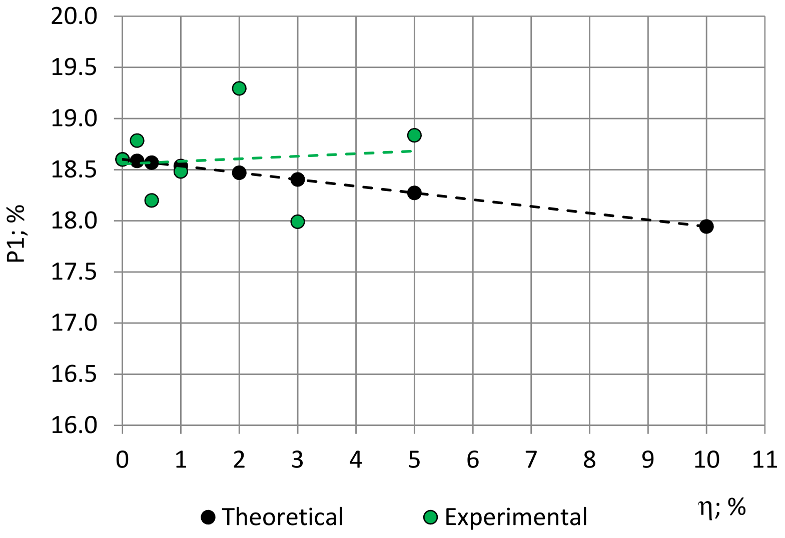

Figure S6. Space filling coefficient P1 of the moulded blocks in dependence of filler's concentration η: Theoretical estimation (black) and Experimental data (green). The dashed lines - the corresponding trendlines.

It can be seen that theoretically P1 decreases linearly with an increase of the concentration of filler η from 18.6 % for neat foams to 18.3 % at 5 % of filler's concentration and porosity P2 increases from 81.4 % to 81.7 %. In limit cases, when η = 0.0 (No filler in the PU foams' formulation), P1 = ρ_{fa} /ρ_{pol} = 18.6 % and when η = 1.0 (Only filler), P1 = ρ_{fa} /ρ_{pol} [1 - η(ρ_{fil} - ρ_{pol})/ρ_{fil}] = 12.0 %. Practically deviations, mentioned in the Point 2.2 of the article, appear in the technological process, leading to a scatter in experimental values of mass and space filling coefficient of PU foams' blocks.

5 Dimensions of circles

The biggest dimensions of typical circles in the printed images were ~ 40 – 45 mm, therefore 5 mm in the printed image were assumed as a nominal unit (5 mm = 1 unit = 0.0286 mm in nature) for division of the selection into classes. Each selection of circles' diameters was divided in i = 1, 2, ..., 10 classes of width one unit. The relative frequency for each class was calculated as:

$$m_i(d) = n_i(d)/N_c, \quad \text{(S3)}$$

where n_i – number of circles in the i-th class and histograms were constructed, Figure S7.

(**a**) (**b**)

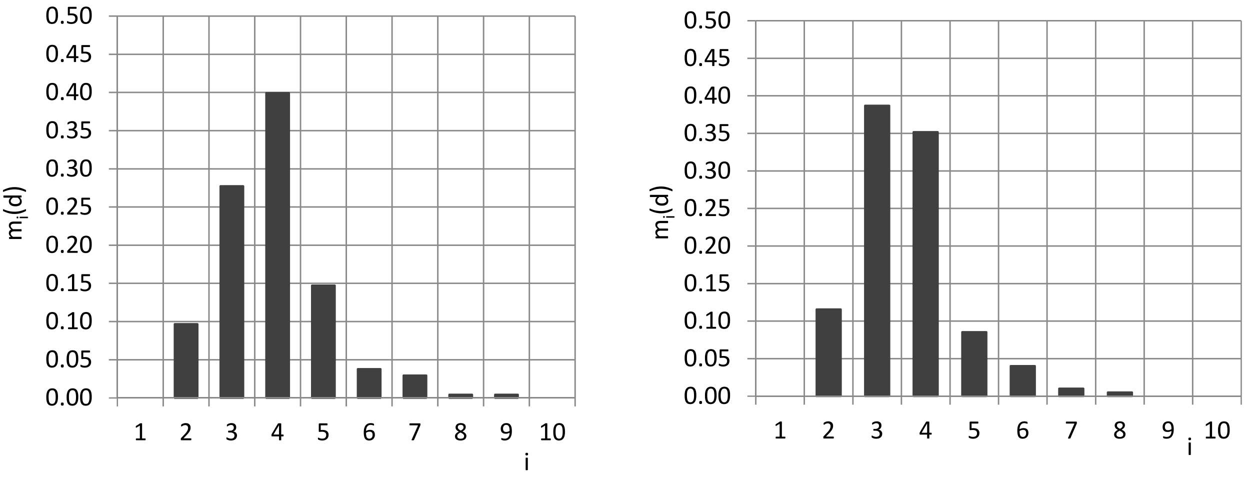

Figure S7. Histograms of circles' diameters of PU foams (**a**) η = 0.0 % and (**b**) η = 5.0 %.

It can be seen that the modal class of the neat PU foams' histogram is the class №4, range 0.086 mm – 0.114 mm. Representing the classes with the central values, the range of variation (Amplitude) of the corresponding selection of circles' diameters is estimated as $d^c_{max} - d^c_{min}$ = 0.20 mm. Modal class of 5% nanoclays' filled PU foams' histogram is the class №3, range 0.057 mm – 0.086 mm, range of variation of the selection is narrower: ≈ 0.17 mm. In the filled PU foams the average diameter of circles d_{aver} ≈ 0.090 mm is ≈ 10 % smaller than in the neat PU foams: d_{aver} ≈ 0.099 mm, meaning that in the filled foams the cells are smaller. The relative frequencies of modal classes are approximately equal in both cases: $m_k(d)$ = 0.40 and $m_k(d)$ = 0.39. The histograms lack elements in the class №1 (Range 0.000 mm – 0.029 mm) suggesting that practically all bubbles have grown above at least the size of diameter ≈ 0.029 mm.

Parameters of the normal PDF-s, approximating the histograms of circles' diameters, were calculated for the 7 microscopy samples, Table S2, taking into account the scale, implemented for division in classes: 5 mm = 1 unit = 0.0286 mm in nature. In Table S2 N_c is the number of circles in the statistical sample, d_{aver} is the average value of diameters d, s - standard deviation of experimental data and v – coefficient of variation. The dimensions of the circles are the smallest at the highest fillers concentration 5 %, confirming the role of filler's particles in nucleation of new bubbles. The relative difference between average diameters of circles of filled and unfilled foams is d_{aver} are ≤ 10 %. It can be seen that the experimental data selections have higher values of coefficient of variation v ≈ 30 – 50 % than the approximating functions, providing values of v ≈ 25 – 30 % due to their smoothing character with regard to the histograms.

Table S2. Distribution characteristics of circles' diameters.

Concen-tration	Circles' diameters (Experimental data)				Probability density function f(d, μ, σ)		
η; %	N_c	d_{aver}; mm	s; mm	v; %	μ; mm	σ; mm	v; %
0.00	250	0.099	0.035	36	0.109	0.029	26
0.25	200	0.091	0.030	33	0.103	0.029	28
0.50	200	0.094	0.032	34	0.103	0.029	28
1.00	250	0.094	0.030	32	0.102	0.034	34
2.00	200	0.091	0.048	53	0.103	0.026	25

3.00	250	0.096	0.039	40	0.106	0.026	24
5.00	250	0.090	0.033	36	0.097	0.026	26

6. Length of struts

Figure S8 comprises histograms of struts' length projections of neat and 5% filled PU foams. In both cases the modal class is the class №3, range 0.0114 mm – 0.0171 mm. The relative frequency of modal class is higher for the filled foams and the filled foams histogram has fewer elements in classes №5 – 8, corresponding to longer dimensions. In both histograms a shortage of elements in the class №1 exists due the problems to identify the short projections on the images (Point 2.4.4 of the article).

(**a**) (**b**)

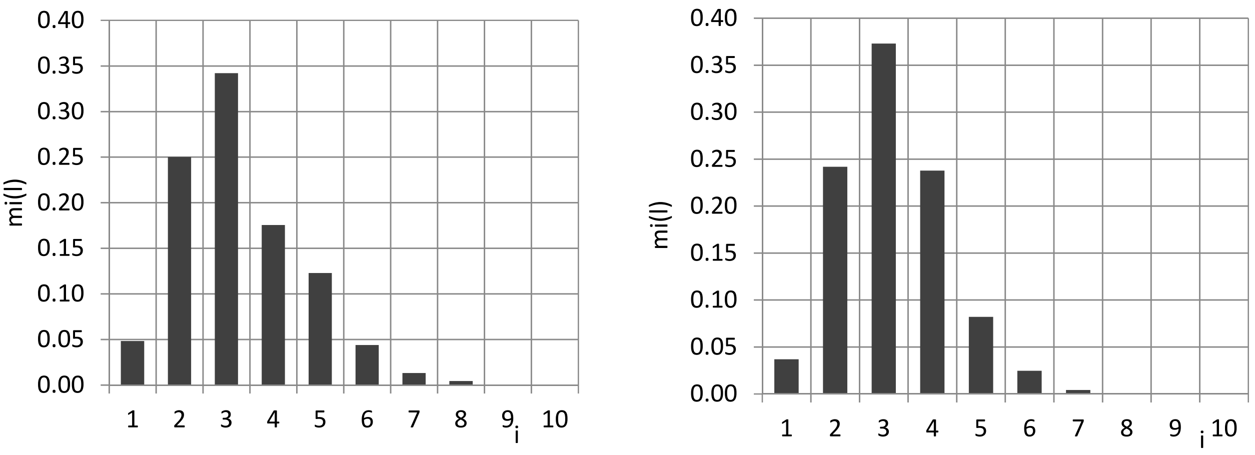

Figure S8. Histograms of struts' length projections of PU foams (**a**) η = 0.0 % and (**b**) η = 5.0 %.

The distribution characteristics of the polymeric struts' length projections' l, both for the experimental data and their approximating functions, as well as distribution characteristics of struts' restored length L are given in Table S3. The largest selections were picked for filler's concentration 3 % and 5 % to ensure proper estimations.

Table S3. Distribution characteristics of struts' length projections l and length L.

η; %	Length projections								Length; functions f(L, q, α, b)			
	Experimental data				Functions f(l, q, α, b)							
	N_{sp}	l_{aver}; mm	s; mm	v; %	α	l_{aver}; mm	σ; mm	v; %	α	L_{aver}; mm	σ; mm	v; %
0.00	250	0.016	0.007	44	0.253	0.016	0.009	56	0.197	0.019	0.011	58
0.25	250	0.018	0.006	33	0.272	0.015	0.008	53	0.195	0.019	0.010	53
0.50	250	0.017	0.006	35	0.357	0.013	0.007	54	0.234	0.017	0.009	53
1.00	250	0.019	0.006	32	0.351	0.013	0.007	54	0.254	0.016	0.009	56
2.00	250	0.015	0.005	33	0.355	0.013	0.007	54	0.279	0.015	0.008	53
3.00	300	0.015	0.006	40	0.327	0.014	0.007	50	0.253	0.016	0.009	56
5.00	300	0.015	0.007	47	0.369	0.012	0.007	58	0.280	0.015	0.008	53

The probability density functions f(l) and f(L) of struts' length projections and struts' length for neat, η = 0 % and for filled, η = 5 %, PU foams are given in Figure S9. It can be seen that filling has narrowed the amplitude of struts' length variation and the struts' average length gradually decreases for 10 – 20 %. The 5 % nanoclays' filled foams have a more uniform cellular structure than the neat foams. At η = 5 % the maximum of struts' length has shifted to the side of smaller values meaning that in the filled foams the struts are shorter.

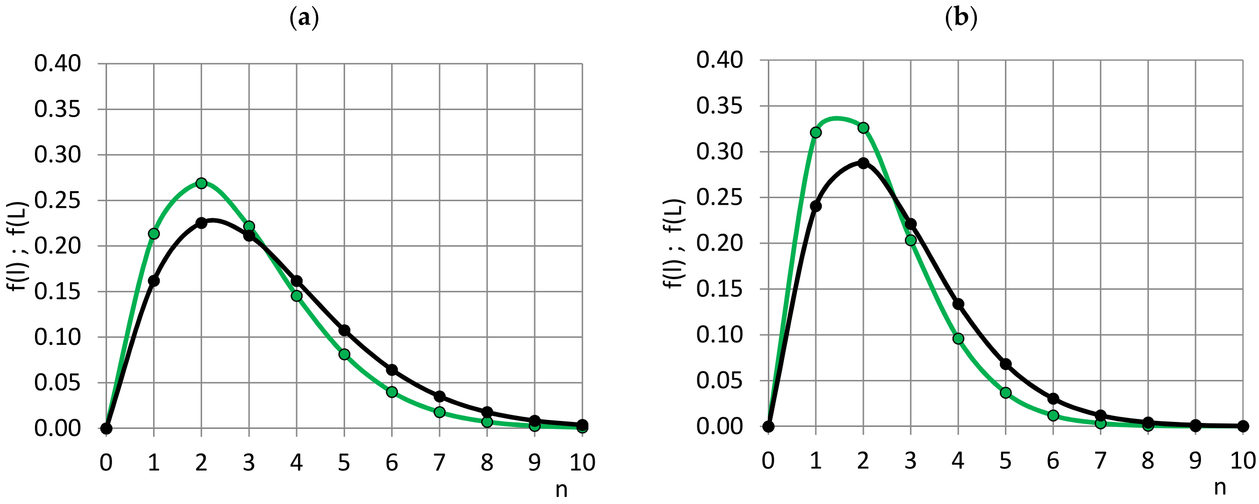

Figure S9. PFD-s of struts' length projections l (Green) and length L (Black) of PU foams; concentration of filler (**a**) η = 0.0 %; and (**b**) η = 5.0 %.

References

1. Beverte, I. Determination of Highly Porous Plastic Foams' Structural Characteristics by Processing LM Images Data. *J. Appl. Polym. Sci.* **2014**, *131*, 39477.
2. Berlin, A.A.; Shutov, F.A. *Chemistry and Technology of Gas-Filled High-Polymers (Химия и Технология Газонаполненных Высокополимеров)*; USSR: Moscow, Russia, 1980; 503p. (In Russian)
3. Renz, R. Zum Zuegigen und Zyklischen Verfomungsverhalten Polymerer Hartschaumstoffe. Ph.D. Thesis, Universität Karlsruhe (TH), Karlsruhe, Germany, 1977. (In German)
4. Zhu, T.; Zhou, C.; Kabwe, F.B.; Wu, Q.; Li, C.S.; Zhang, J.R. Exfoliation of montmorillonite and related properties of clay/polymer nanocomposites. *Appl. Clay Sci.* **2019**, *169*, 48–66. https://doi.org/10.1016/j.clay.2018.12.006.
5. Kirpļuks, M. Development of Renewable Feedstock Based Rigid Polyurethane Foam and Nanoclay Composites. Ph.D. Thesis, Riga Technical University, Riga, Latvia, 2020. Available online: https://ortus.rtu.lv/science/en/publications/31088 (accessed on 15 November 2021).
